# Novel 3D video action recognition deep learning approach for near real time epileptic seizure classification

**DOI:** 10.1038/s41598-022-23133-9

**Published:** 2022-11-15

**Authors:** Tamás Karácsony, Anna Mira Loesch-Biffar, Christian Vollmar, Jan Rémi, Soheyl Noachtar, João Paulo Silva Cunha

**Affiliations:** 1 Center for Biomedical Engineering Research, Institute for Systems’ Engineering and Computers, Technology and Science (INESC TEC), Porto, Portugal; 2 Epilepsy Center, Department of Neurology, University of Munich, Munich, Germany; 3 Faculty of Engineering (FEUP), University of Porto, Porto, Portugal

**Keywords:** Computational neuroscience, Biomedical engineering, Computational science, Translational research, Epilepsy

## Abstract

Seizure semiology is a well-established method to classify epileptic seizure types, but requires a significant amount of resources as long-term Video-EEG monitoring needs to be visually analyzed. Therefore, computer vision based diagnosis support tools are a promising approach. In this article, we utilize infrared (IR) and depth (3D) videos to show the feasibility of a 24/7 novel object and action recognition based deep learning (DL) monitoring system to differentiate between epileptic seizures in frontal lobe epilepsy (FLE), temporal lobe epilepsy (TLE) and non-epileptic events. Based on the largest 3Dvideo-EEG database in the world (115 seizures/+680,000 video-frames/427GB), we achieved a promising cross-subject validation f1-score of 0.833±0.061 for the 2 class (FLE vs. TLE) and 0.763 ± 0.083 for the 3 class (FLE vs. TLE vs. non-epileptic) case, from 2 s samples, with an automated semi-specialized depth (Acc.95.65%) and Mask R-CNN (Acc.96.52%) based cropping pipeline to pre-process the videos, enabling a near-real-time seizure type detection and classification tool. Our results demonstrate the feasibility of our novel DL approach to support 24/7 epilepsy monitoring, outperforming all previously published methods.

## Introduction

Epilepsy is a very common chronic neurological disease and affects 1% of the population worldwide^1^. Seizures are the defining symptom, and their form (semiology) is paramount for differential diagnosis and localization of the seizure onset zone in the brain^2^. This is especially important for pharmacoresistant epilepsy patients considered for epilepsy surgery. Currently, seizure analysis is based on the visual interpretation of 2D video-EEG data in epilepsy monitoring units (EMUs) by highly specialized clinicians^3,4^, where semiology evaluation is limited by a high inter-rater variability^5^. Automated and semi-automated computer-vision analysis approaches have been reported as promising in the literature^6^, but still depend on considerable “human in the loop”^7^ effort.

**Table 1:** Results and comparison.

Author	Classes	Performance (cross subject, sequence wise)	Notes
Achilles et al.^8^	Seizure No seizure	AUC: 0.78	Single frame approach (posture recognition)
Ahmedt-Aristizabal et al.^9^	MTLE ETLE	Average accuracy: 56.31% (best, just body)	Face body and hand inputs, very high std
Ahmedt-Aristizabal et al.^10–12^	MTLE ETLE	Average accuracy:50.85%^10^; 58.49%^11^; 69.8%^12^	Subject specific accuracy 95.19%^10^; 92.10%^11^; 89%^12^; susceptible to overfit to subject specific facial features^10–12^ and posture coordinates^11^
Ahmedt-Aristizabal et al.^13^	MTLE ETLE	Average accuracy: 66.48%; 62.19%	Promising aggregated cosine similarity results through seizures AUC: 0.9703
Maia et al.^14^	TLE ETLE	AUC 0.65	Probably overfits
Karácsony et al.^15^	TLE FLE	f1-score: 0.844±0.042 (AUC: 0.90±0.04)	2 class
**This work**	**TLE** **FLE**	**f1-score:** ** 0.833**±**0.061** ** (AUC: 0.89**±**0.08)**	**2 class**
	**TLE** **FLE** **Prepost**	**f1-score:** **0.763**±**0.083**	**3 class**

(a) Comparison to other deep learning based publications in this domain.

(*ETLE* extra temporal lobe epilepsy, *MTLE* mesial temporal lobe epilepsy, *std* standard deviation).

Despite a vast amount of video material available, quantitative seizure classification studies are still rare^16,17^. Even more rare are approaches for automated, AI-supported solutions (Table 1). We previously proposed a convolutional neural network (CNN) based epilepsy classification with IR and depth video input^8,18^. Utilizing the Neurokinect 3D video dataset^19–21^—to the best of our knowledge the largest 3D-video-EEG database in the world–we combined Inception-V3 feature extraction and a fully connected classifier, to process IR seizure videos, achieving a modest result (AUC 0.65)^14^. We postulate that these modest results were due to the lack of temporal information of the object recognition training of the classifier and that it may have been influenced by class imbalance and overfit to one of them. Other studies used a hierarchical approach^9,22^, processing three main parallel threads of body regions and posture^10–12^. Accuracy was high when training and validation used the same subjects, but the “leave one subject out” cross validation yielded only modest accuracy (50.9–69.8%)^10–12^, suggesting the inability to capture subject invariant features and subsequent overfit to subject specific facial features and posture coordinates. A shallow CNN and long short-term memory (LSTM) based architecture was also used in the literature, but no major improvement was obtained (62.2—66.5%)^13^.

In this paper we present a novel contribution inspired by the way epileptologists analyse seizure semiology where they take into account, not only with the presence of specific “Movements Of Interest” (MOI) in different parts of the patients’ body, but also its dynamics (the sequence of their appearance) and their biomechanics characteristics (e.g. speed/acceleration patterns, movement amplitude, etc.). Thus, we decided to explore the inclusion of these spatio-temporal aspects by developing a novel DL action recognition approach and study its feasibility for a 3 class general, cross-subject, near-real time epileptic seizure classification pipeline for 24/7 automated seizure detection at the EMUs.

## Results

Our DL seizure classification pipeline (Fig. 3) was evaluated using 3 different classification architectures: (1) I3D, (2) LSTM and (3) Extended LSTM, where (1) is the original classification layers retrained, (2) and (3) are well known approaches to exploit long term temporal features even more. These architectures were trained on the I3D features extracted from three datasets and evaluated for the 2 class (FLE, TLE) and 3 class (FLE, TLE, non-epileptic) scenarios.

### Classification of I3D features

**Figure 1:**
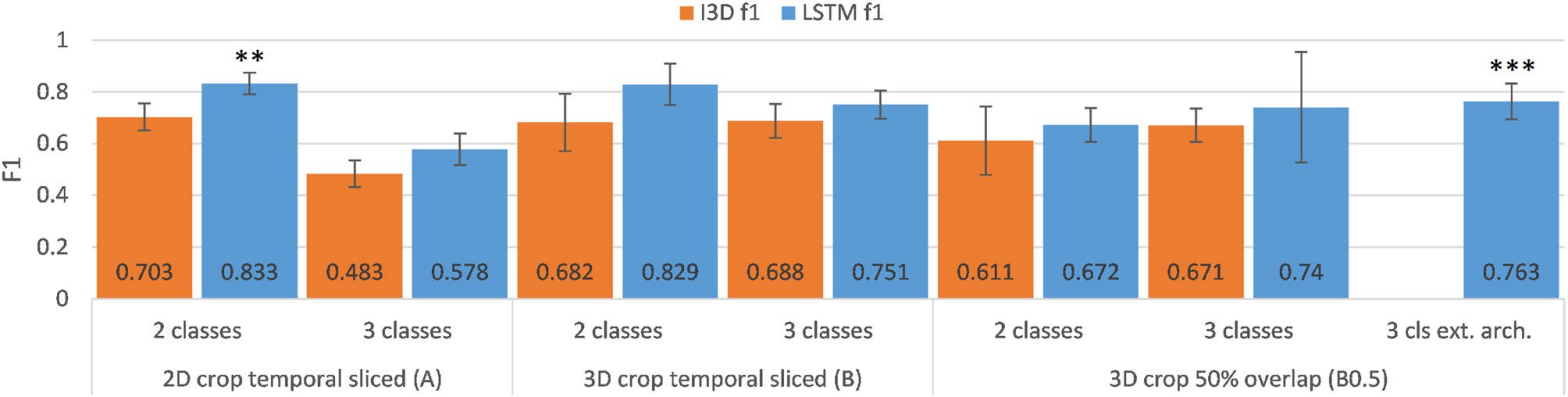
I3D classifier vs. LSTM classifier (see “Implemented classifier architectures”) 5-fold cross validation F1 classification performance comparison for the three datasets. (** best for 2-class differentiation, *** best for 3-class differentiation).

The I3D feature extraction from three datasets was required in order to evaluate the effectiveness of depth cropping and temporal slicing strategies and their effect on classification performance. With the purpose of observing the effectiveness of the depth cropping the “2D crop temporal sliced (A)”,—which only employs the Mask R-CNN cropping,—and the “3D crop temporal sliced (B)”,—which additionally utilizes the depth cropping,—datasets were compared. Moreover to investigate a temporal augmentation strategy the “3D crop overlapping (B0.5)” dataset was evaluated as well, which has 50% overlap between the 2 s samples. (For further details about the datasets see “Data pre-processing”). In the following sections the classification results are presented (Fig. 1).

**Table 2:** 5-fold cross validation F1 scores, sensitivity (Sens.) and specificity (Spec.) results for all 3 datasets and designed architectures, (*cls* number of classes, *ext. LSTM* extended LSTM archtiecture).

Dataset	cls	I3D f1	LSTM f1		I3D Spec.	I3D Sens.	LSTM Spec.	LSTM Sens.
2D crop temporal sliced (A)	**2**	0.703 ± 0.053	***0.833 ± 0.061***	FLE	0.747 ± 0.154	0.695 ± 0.164	**0.870 ± 0.041**	**0.794 ± 0.157**
				TLE	0.695 ± 0.164	0.747 ± 0.154	**0.794 ± 0.157**	**0.870 ± 0.041**
	3	0.483 ± 0.051	0.578 ± 0.080	FLE	0.854 ± 0.044	0.592 ± 0.116	0.942 ± 0.026	0.444 ± 0.134
				TLE	0.795 ± 0.062	0.400 ± 0.064	0.902 ± 0.074	0.426 ± 0.114
				Non-epileptic	0.610 ± 0.107	0.635 ± 0.094	0.501 ± 0.064	0.838 ± 0.090
3D crop temporal sliced (B)	2	0.682 ± 0.111	0.829 ± 0.055	FLE	0.646 ± 0.115	0.771 ± 0.132	0.886 ± 0.064	0.764 ± 0.152
				TLE	0.771 ± 0.132	0.646 ± 0.115	0.764 ± 0.152	0.886 ± 0.064
	3	0.688 ± 0.066	0.751 ± 0.065	FLE	0.886 ± 0.024	0.648 ± 0.178	0.941 ± 0.032	0.613 ± 0.203
				TLE	0.941 ± 0.036	0.637 ± 0.109	0.956 ± 0.040	0.720 ± 0.128
				Non-epileptic	0.936 ± 0.025	0.904 ± 0.026	0.873 ± 0.043	0.949 ± 0.032
3D crop w/ 50% overlap (B0.5)	2	0.611 ± 0.133	0.672 ± 0.214	FLE	0.621 ± 0.113	0.663 ± 0.217	0.953 ± 0.041	0.461 ± 0.330
				TLE	0.663 ± 0.217	0.621 ± 0.113	0.461 ± 0.330	0.953 ± 0.041
	3	0.671 ± 0.065	0.740 ± 0.069	FLE	0.891 ± 0.027	0.619 ± 0.178	0.921 ± 0.031	0.648 ± 0.189
				TLE	0.922 ± 0.040	0.645 ± 0.087	0.970 ± 0.015	0.672 ± 0.148
				Non-epileptic	0.935 ± 0.022	0.887 ± 0.026	0.910 ± 0.045	0.958 ± 0.014
	**cls**	**I3D f1**	**Ext. LSTM f1**		**Ext. LSTM arch. Spec**.		**Ext. LSTM arch. Sens.**	
	**3**	-	***0.763 ± 0.083***	FLE	**0.934 ± 0.022**		**0.639 ± 0.215**	
				TLE	**0.962 ± 0.029**		**0.765 ± 0.058**	
				Non-epileptic	**0.922 ± 0.024**		**0.947 ± 0.019**	

Best results are in bold.

The performance of the classification of I3D features were evaluated with the macro averages of the 5-fold cross-validation metrics. This approach handles all classes with the same weight, thus eliminating the effect of the class imbalance. It maximizes the f1-score, precision, recall and other metrics for all classes at the same time. It prevents overfitting for the class with more samples, which may lead to false suggestions of higher global performance, by disregarding the minority class^23^. In the following sections the average ± standard deviation of the 5-fold cross validation macro average metrics f1-score are going to be reported for the developed best performing approaches (Table 2).

#### LSTM classifier

The hyperparameter search of the LSTM classifier pointed to the same hyperparameters, except for dataset B0.5 where an extended architecture still achieved minor improvements (see detailed hyperparameters in “Implemented classifier architectures”).

Concerning the 2 class case (Fig. 1) the simplest preprocessing, utilizing only the 2D cropping (A), performed the best with an F1 score of 0.833 ± 0.061. The additional depth cropping (B) did not have substantial effect but on the contrary, the temporal overlapping strategy (B0.5) decreased dissimilarity of the samples and thus increased generalization errors.

Regarding the 3 class case (Fig. 1), the substantially larger 3 class dataset B and B0.5, with additional depth cropping and temporal augmentation, considerably increased the performance. The 3D cropping strategy (B), compared with dataset A significantly improved F1 score with 0.173. Furthermore, on dataset B0.5, an extended architecture containing more neurons in the classification layers (see “Implemented classifier architectures”) achieved some additional improvements and achieved an F1 score of 0.763 ± 0.083.

#### I3D classifier

The originally described I3D network with the retrained classifier layer (Fig. 3) best performed in the 2 class case on the 3D cropped dataset B. The temporal overlapping dataset B0.5 performed worse than dataset B (Fig. 1). In this case the additional depth cropping improved the performance, however the temporal overlapping slicing reduced performance, due to the small dataset size this augmentation did not break correlation enough, thus overfitting the data.

Regarding the 3 class case (Fig. 1) the performance followed the pattern, dataset B performed the best and temporal augmentation did not introduce improvements. It should be underlined that the 3D cropping (B) compared with the 2D cropping (A) improved the f1 score substantially, with more than 0.2.

### Pre-processing

**Figure 2:**
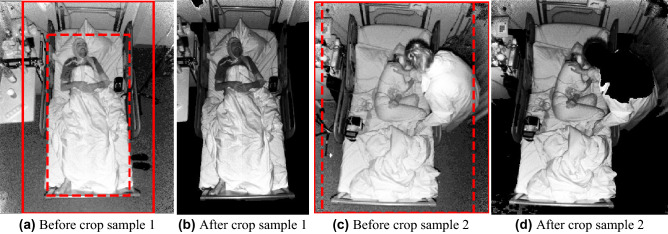
Examples of detection and crop, when the bed and the patient were properly detected, surrounding scenery removed, with enough space left to capture the full scale of seizures (**a**,**b** 2D Mask R-CNN crop is more effective, **c**,**d** depth crop is more effective, dotted red line—detection box, straight red line—crop box).

The results of the two cropping approaches—Depth and Mask R-CNN video cropping - were evaluated by visual confirmation of the videos (Fig. 2). The evaluation included 5 frames from each video distributed evenly through the length of of the sequence including the first and the last frames.

#### Depth cropping results

After adequate denoising, the depth algorithm properly cropped 110/115 seizures (95.65%). The five exceptions had abnormally high scene complexity, such as three clinicians attending to the patient and blocking the camera on the evaluated frame. These seizures were manually cropped with adequate depth boundaries for the next steps.

The cropped depth videos were then used to crop the IR videos, which adequately removed the surrounding scenery, occlusions between the camera and the patient, such as clinical personnel (Fig. 2d), and most of the background. However as the algorithm is utilizing two flat planes to crop the scene some of the scenery on the sides in the depth range between the two planes remained, which were addressed with Mask R-CNN cropping for the final 3D cropped dataset B and B0.5.

**Figure 3:**
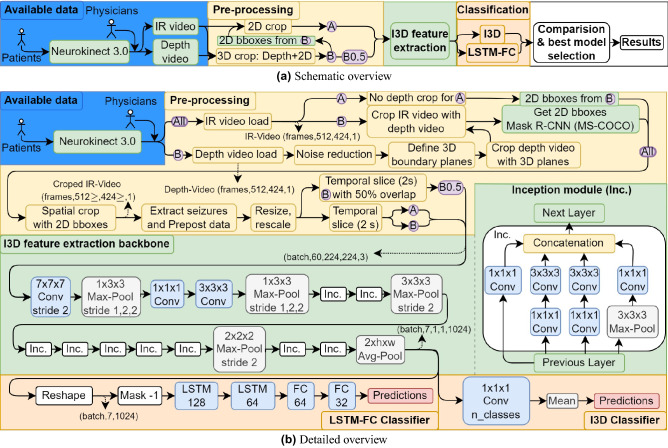
(**a**) Schematic overview of the architecture pipeline, and (**b**) detailed overview including the available data from Neurokinect 3.0^21^, the pre-processing algorithm for all 3 datasets, (A–2D (Mask R-CNN) cropping with bounding boxes of B; B–2D and depth cropping; B0.5–B with 50% overlapping 2s temporal slices, see “Data pre-processing”), the I3D feature extraction backbone^24^ (see “Feature extraction”) and the LSTM-FC and I3D classifiers (see “Implemented classifier architectures”). (Figure extended from our previous work^15^).

#### 2D Mask R-CNN video cropping

The results of the bounding box detection with Mask R-CNN were also visually confirmed (see “Pre-processing”), as there were no available predefined bounding boxes.

The designed algorithm successfully detected on the depth cropped videos and cropped the area of interest in 111/115, 96.52% of the cases (Fig. 2). A +30% expansion of the detected bounding box was applied to ensure complete capture of the full seizure, including extensive movements. This expansion of the cropping box in turn included some of the surroundings (Fig. 2). Nevertheless, the algorithm properly removed most of the remaining background and unnecessary surrounding scenery.

Only in four out of the 115 cases (3.47 %) detection was not satisfyingly precise, namely not including the full patient. This under- or misdetections of the region of interest was due to heavy occlusions from the environment both on the patient and the patients’ bed (1 case), sometimes combined with a position of the patient and angle of view with moderate self occlusions (3 cases). These 4 seizure videos were replaced with manually reannotated and cropped ones for the feature extraction phase. On the other hand, we must emphasize that in several other instances the algorithm handled similar scenarios well, such as physicians on the frame, sitting position of the patient or partial occlusions on the bed and patient. Moreover the combined depth and Mask R-CNN cropping (3D crop) produced good results in spite of the complex scenery with occlusions, such as the blanket or the surrounding medical staff.

The developed depth and Mask R-CNN based 3D cropping method is flexible and can be applied for different EMU setups, regardless of angle of view, orientation and distance to the camera.

## Discussion

In EMUs, physicians depend on subjective evaluation of video-EEG data and the continuous monitoring of patients on these highly specialized medical units requires significant resources. Machine learning approaches may be of great value, to accelerate the processing of huge amounts of data, assist in detecting and classifying seizures, reducing inter-rater variability, and ultimately improve syndrome diagnosis^25^. Therefore, the application of recent developments in the field of computer vision for movement based epilepsy classification is an encouraging, however still not widely utilized approach, mostly due to the limitations of available data.

Our Neurokinect dataset^19–21^ is unique for its depth and the IR videos of epileptic seizures, and combined with the clinical experience of the Epilepsy Center Munich, it provides a great source for labelled clinical data for supervised machine learning. Although for data intensive approaches as DL it is still limited in size and imbalanced between classes, it provides a very good basis for research and developing proof-of-concept systems. The limitations of the dataset can be mitigated by taking advantage of transfer learning from other large datasets. Nevertheless, to the best of our knowledge, we present here the first ever 3D-video-based DL approach for near-real-time epilepsy seizure detection and classification.

### Performance of preprocessing

Our preprocessing algorithm is semi-specialized and automatic, which significantly speeds up the removal of unnecessary surroundings. The depth cropping and Mask R-CNN based 3D cropping complement each other, as it is illustrated on Fig. 2. With only limited occlusion of the scenery, Mask R-CNN alone already performs adequate cropping, and depth cropping further improves the scenery. On the other hand, when there are several occlusions in a challenging scenario, depth cropping can remove objects between the camera and the patient, such as clinical personnel as seen on sample 2 (Fig. 2d). The combination of the two cropping methods provides a generally cleaned scenery. Thus, to extract relevant information from the available videos and minimize unrelated variations, our automated 3D cropping algorithm based on the Mask R-CNN with successful cropping rate of 96.83% and the depth cropping with 95.65% successful cropping rate are very encouraging results. In the future, the algorithm might be still improved, by evaluating and cropping each frame individually, as it would crop more closely and would not require the 30% buffer extension of the bounding box to ensure the inclusion of movements with large extent. However, that approach would require significantly more model inference, thus computation time, and it might not improve results greatly.

### Effect of pre-processing on classification performance

#### Depth cropping: comparing A to B

The introduction of the depth cropping preprocessing technique in the 3 class case significantly increased performance for both the I3D and LSTM based classifiers. For the I3D classifier it improved performance from 0.483 to 0.688 with 0.205 absolute f1 score improvement and for the LSTM it improved this metric from 0.578 to 0.751 (+0.173). In dataset A, with 2D cropping only, there is significant confusion to the prepost class (See in the additional material Fig. A.4), mostly because the feature extraction might pick up movements around bed as well, not related to the seizure, thus introducing noise to the classifier. Utilizing the additional 3D cropping technique for dataset B, removes this unrelated movement around the bed and between the camera and the patient, thereby excluding it from the feature extraction, reducing confusion and improving the classification In the 2 class case there is no significant difference between the performance of dataset A and B, even slight performance drop for both I3D and LSTM classifiers, it improved confusion for some cross validation runs, however it decreased for others.

Depth cropping impacted the 3 class case significantly more than the 2 class case. In the 3 class case there is a significantly larger dataset available, especially for the 3rd non seizure class, therefore the noise reduction has considerably more positive impact. In contrast the 2 class, with less data, which is already more curated, the reduction of fluctuations does not introduce such improvements. This can be observed in the additional material on Figs. A.1 vs. A.2 for the 2 class, and Figs. A.3 vs. A.4 for the 3 class case.

The Prepost data has a lot more heavy occlusions due to two reasons. In this class, especially the post seizure part, there is almost always clinical staff present, taking care of the patient, applying medication, repositioning the patient, etc. These activities, without depth cropping, can be included into the feature extraction.

#### Temporal augmentation: comparing B to B0.5

The additional temporal augmentation slightly increased the 3 class LSTM performance when the architecture was extended in dataset B0.5 compared to B. On the standard LSTM architecture it generally improved performance in 4 out of 5 cross validation runs, however it overfit in one run, which decreased the average metric.

On the other hand, for the 2 class LSTM and I3D architectures it decreased the performance. By analysis of the confusion matrices and training, rapid overfit was observed to class 1 (TLE). As this class has less than half the number of seizures than class 2 (FLE), but the same number of samples, it presents less variation between samples. Therefore the weighted loss and other regularization techniques were not able to counterbalance this over correlated class enough, as temporal slicing is already just an augmentation, not new data introduction. This might be counteracted with an additional weighting of samples, although there is a significant variation of samples and MOI representation between cross validation runs, which would make it essentially an extra hyperparameter for now, as this MOI representation is not available yet in the dataset. It should be noted that a one frame sliding window dataset was tested as well, which produced the same pattern, with overfitting in less than an epoch. Additionally stronger augmentations could be tested, but those might compromise epilepsy related information. Thus, this analysis points out that to substantially improve performance a lot more data will be required, preferably in a more balanced manner between classes. All that said the feasibility of such a system is proven, even with a limited and imbalanced dataset.

#### 2 sec slicing strategy

A significant contribution of our work is proving the feasibility of the proposed temporal slicing strategy, using 2 s samples to differentiate between the seizures. It establishes that sufficient information can be extracted utilizing an action recognition approach, because it accounts not only for posture, but for the recognition of seizure-related MOIs, mimicking how epileptologist analyse seizure semiology. On the other hand, it also limits the maximal performance of the classifier, as there might be samples, where there are no distinguishable features of the movement during the course of the seizure. This approach therefore is a trade off between the maximal performance of the classifier and the best utilization of the limited clinical data for training. In fact this splitting strategy improved the handling of class imbalance. The seizures for each training originate only from 12 FLE and 8 TLE patients with approximately 62.4 ± 8.19 and 26 ± 2.83 seizures per class respectively and validated from 3 FLE and 3 TLE patients with approximately 15.6 ± 8.19 and 11 ± 2.83 seizures per class respectively. However, the number of seizures available varies per patients. Utilizing the splitting strategy, this resulted in a more balanced distribution between the TLE and FLE classes (Table 3a), due to shorter duration of FLE seizures.

### Comparison of classifiers

In comparison of the I3D and LSTM classifier architectures the developed LSTM performs significantly better in all of the investigated cases (Fig. 1), as it takes into account and utilizes the temporal information even more than the I3D classifier.

The LSTM classifier also has significantly more trainable parameters (see “Methods”), moreover it was specially developed and trained for this dataset. Additionally as the I3D classification layer has less parameters it depends on the preceding feature extraction layers to a larger degree. Therefore unlocking more layers in the training state, or even fine training the whole architecture might improve performance of the I3D as well. Even though the I3D network was originally trained on RGB videos, not on IR, with different movement classes, the transfer learning approach performed well, especially utilizing it as a feature extractor network. The incorporation of relevant information from other significantly larger datasets, by transferring pre-trained weights from similar object and action recognition domains was essential to extract movement features, thus utilizing scarce clinical data only to train the clinical classification. Therefore, the I3D network, which was developed for human action recognition fitted smoothly into our proposed architecture.

In order to improve the feature extraction, the weights of the I3D network could be fine tuned, with the current seizure datasets. Moreover, fine tuning the weights would also improve the distinction of seizure specific movements. The extended LSTM architecture shows that with more data classification performance could be improved, as in the other datasets (A, B, and 2 class B.05) the main limiting factor is the limited data with high variability, thus the extended architecture would easily overfit to the training data, as uncovered by the random hyperparameter search.

### Comparison to the state of the art

Epileptic seizures MOI dynamics matter in the clinical evaluation and our results show that incorporating this temporal dynamic into the DL approach by transfer learning and action recognition embedding, we can improve largely the classification algorithm. To the best of our knowledge, the current work outperforms all previous deep learning based approaches to automated epileptic seizure detection and classification both in performance and generalization of the results Table 1a. Only our previously presented approach^15^ had slightly better f1 score, as it utilized 11 more seizures. Since it surpasses previous publications which either did not use feature extraction with pre-training,—thus significantly sacrificing performance due to lack of data—, or used pre-trained ones for static image recognition^9,14^, which essentially neglect temporal features. For instance, compared to utilizing a single frame approach^8^, a more complex classification problem was used (FLE vs. TLE), instead of just the detection of the presence or absence of seizures. Even with these more specific classes, the developed architecture improved the average AUC with 0.11 to 0.89 ± 0.08, moreover based on the ROC curve^8^ with the 0.870 specificity, the sensitivity was significantly improved with approximately 0.3–0.794. Other approaches which use a simpler binary classification of MTLE vs. ETLE and TLE vs. ETLE classes respectively^9,14^, achieved very limited performances compared to the architecture presented here. The results indicate it is possible to achieve high cross subject performance from 2 s video segments, which were not achieved before^10–13^.

Therefore the developed classification pipeline performed well above the performances reported in the literature, in terms of f1 score, and cross subject generalization, with high specificity and sensitivity for the 2 class case and even higher specificity with a decent sensitivity for the 3 class case. The classifiers managed to handle the considerably limited and imbalanced dataset, with application of transfer learning, regularization techniques and splitting the data into 2 s samples. Compared to our previous contribution^15^ we extended the architecture to 3 classes including the non seizure class. Moreover, we introduced a new preprocessing pipeline, utilizing depth cropping, which considerably improves classification performance.

## Conclusion

We demonstrate a novel state of the art deep learning based approach for motion based 3 class classification of seizures in frontal and temporal lobe epilepsies and a non seizure class. The system uses 3D videos (IR and depth) of the seizures that are 24/7 acquired at the EMUs.

The developed pipeline has 2 components: (1) an “intelligent” cropping and (2) a novel action-recognition classifier. For implementing (1) we combined Mask R-CNN and depth cropping based pre-processing, with a 96.52% and 95.65% successful crop rate respectively. The introduction of 3D depth cropping to remove occlusions and unrelated information from the scene significantly improved classification performance.

For implementing (2), we used I3D feature extraction, LSTM-FC and I3D classification, heavily utilizing transfer learning from static object detection and dynamic human action recognition datasets with network architectures available in the literature, and the best architecture achieved a general, cross subject 5-fold cross-validation f1-score of 0.833 ± 0.061 for the 2 class binary seizure classification and 0.763 ± 0.083 for the 3 class case. To the best of our knowledge, it outperforms all previous deep learning based approaches to video-based seizure classification, indicating a high potential to support physicians with diagnostic decisions.

Moreover the research shows the feasibility of our action recognition approach to distinguish these three classes with only 2 s samples. It evaluated further temporal augmentation techniques, which suggest that larger datasets might benefit more from such augmentation, but in this case it compromises generalization, thus performance.

Our results also shows future potential for online event-detection applications in epilepsy monitoring units, especially the 3 class architecture, which showed a high-potential to be used for near-real-time classification and alarm of seizures in hospitals. Moreover, the designed architecture may be transferred to other 3D video datasets and applications such as ambulant monitoring with minor adjustments.

## Methods

For the purpose of this study, patients’ seizures in the EMU of the University of Munich Epilepsy Center were recorded and analyzed. The ultimate goal in automated seizure analysis is the differentiation of various epilepsy syndromes and their non-epileptic differential diagnoses by their seizures/attacks alone, which is difficult to perform with high accuracy on clinical grounds alone and hampered by high inter-rater variability, as mentioned above. We therefore defined three classes along typical clinical use cases, namely seizures in frontal lobe epilepsy (FLE, class 1), temporal lobe epilepsy (TLE, class 2), and non-epileptic movements of the pre- and postictal parts of the recordings (Prepost, class 3). Ground truth definition was based on all available data (semiology, EEG, MRI, SPECT/PET, neuropsychology) and defined in interdisciplinary patient management meetings. The extent, frequency, amplitude and duration of the movements in these classes are clinically different but with low separation power on clinical grounds alone.

### Data acquisition

The dataset was acquired with the NeuroKinect 3.0 system implemented at the EMU of the University of Munich^20,21^. This system is a three-bed Kinect v2 3Dvideo-EEG system developed for epileptic seizure monitoring. Kinect v2 acquires multiple streams of data namely, 1920 $\times$ 1080 HD-RGB, 512 $\times$ 424 infrared (IR) and depth videos, and 3D body joint information, with a sampling rate of 30 fps.

All methods were carried out in accordance with relevant guidelines and regulations, all experimental protocols were approved by the Ethical Commission of Ludwig Maximilian University of Munich with project number 217-13. Informed consent was obtained from all subjects, including publication of images from them.

**Table 3:** Dataset.

**(a) Main patient demographics and metrics of the extracted IR and depth videos dataset**				
Class name	FLE	TLE	Prepost	Total
Included seizures	FLE, right FLE,left FLE	TLE, right TLE,left TLE	Pre-ictal,Post-ictal	FLE,TLE, Prepost
Number of patients	15	11	26	
Average age	32.44 ± 9.85	38.81 ± 19.20	35.03 ± 14.75	
Number of seizures	78	37	115	
Total length [frames]	73,444	81,592	531,628	686,664
Average video length [frames]	941.6	2205.2	4663.4	2998.5
Minimal video length [frames]	179	661	39	39
Maximal video length [frames]	5636	6779	27293	27293
60 frame samples temporal slicing	1252	1372	8852	11476
60 frame samples temporal overlapping	2333	2666	17424	22423
Data stream channels	IR and depth			
Resolution	512 $\times$ 424 16bit each channel			
Kinect sampling frequency	30 fps			
Dataset total size	427 GB			

**(b) Summary of sub-datasets by preprocessing**				
Identifier	Mask R-CNN cropping	Depth cropping	Temporal augmentation	
A	Yes	No	60 frame slicing	
B	Yes	Yes	60 frame slicing	
B0.5	Yes	Yes	60 frame slicing with 50% overlap	

(*FLE* frontal lobe epilepsy, *TLE* temporal lobe epilepsy, *Prepost* pre- and postictal parts of the videos.)

### Extracted dataset

As a next step, IR and depth videos were extracted for this study, as the proposed pipeline is intended to be used for 24/7 (day and night) for near-online monitoring. The main metrics of the extracted dataset are described in Table 3a and the seizures occurred per patients in Table 4.

This dataset is based on the data, that we previously described^15^, now augmented with the third, non-epileptic class, totaling 427 GB of data.

### Data pre-processing

#### Depth video noise reduction

The depth stream recorded by the Kinect v2 contains zero value pepper noise, thus a morphological dilation had to be applied to close these missing pixel values, as they would have been translated to the IR stream with the depth based cropping. Frames were dilated with a 4 $\times$ 4 rectangular structuring element for 2 iterations. This adequately removed the pepper noise in preparation for cropping the IR videos.

#### Spatial cropping of videos

Depth and video-bounding-box cropping was performed to focus the field of interest, remove unrelated movements, essentially noise from the scenery.

Depth cropping

**Table 4:** Detailed clinical information of the patients and seizures in the dataset. (P. # - Patient Id; # of sz - Number of used seizures, Inv. rec. - Recordings with invasive Electrodes; f - female, m - male, sz. - seizure)

P. #	Sex	# of sz	Syndrome	Etiology	Inv. rec.	Seizure types of seizures in the dataset	Overall semiologies per patient
1	m	16	Left FLE	Unknown	Yes	1. Complex motor sz.	1. Hyponpompic sz. $\rightarrow$ complex motor/hyperkinetic sz.$\rightarrow$ GTC
						2. Hyperkinetic sz.	
						3. Hyponpompic sz.	
2	f	3	Left TLE	Sclerosis of left hippocampus	No	1. Automotor sz.	1. Automotor sz. $\rightarrow$ tonic sz. of the left arm $\rightarrow$ right versive sz. $\rightarrow$ GTC
3	m	5	Right FLE	Right frontal hamartoma	Yes	1. Hyperkinetic sz.	1. Hyperkinetic sz. $\rightarrow$ GTC
						2. Hyperkinetic sz. $\rightarrow$ GTC	
4	m	1	Right FLE	Unknown	Yes	1. Bilateral tonic sz. $\rightarrow$ automotor sz.	2. Epigastric aura $\rightarrow$ dialeptic sz. $\rightarrow$ generalized tonic sz. $\rightarrow$ automotor sz. $\rightarrow$ clonic sz. of the right face $\rightarrow$ GTC
5	m	1	Left FLE	Unknown	No	1. GTC	1. Dialeptic sz./automotor sz. $\rightarrow$ right versive sz. $\rightarrow$ right clonic sz. $\rightarrow$ GTC
6	f	2	Right FLE	Unknown	No	1. Left versive sz. $\rightarrow$ GTC	1. Dialeptic sz. $\rightarrow$ left versive sz. $\rightarrow$ GTC
						2. Left versive sz.	
7	f	4	Left FLE	Left frontal cortical dysplasia	No	1. Right versive sz. $\rightarrow$ clonic sz. of the right face/arm	1. Right versive sz. $\rightarrow$ clonic sz. of the right face $\rightarrow$ GTC
						2. Right versive sz. $\rightarrow$ GTC	2. Hyponpompic sz.
8	f	1	Left FLE	Perinatal defect on the left	No	1. Hyponpompic sz.	1. Tonic sz. of the right hand $\rightarrow$ GTC
							2. Hyponpompic sz.
9	m	1	Left FLE	Unknown	No	1. Automotor sz.	1. Right versive sz. $\rightarrow$ GTC
							2. Automotor sz.
10	m	7	FLE	Left frontal cortical dysplasia	No	1. Complex motor sz.	1. Complex motor sz.
						2. Hyponpompic sz.	2. Hyponpompic sz.
11	m	2	Right FLE	Unknown	No	1. Left clonic sz.	1. Left clonic sz. $\rightarrow$ left versive sz. $\rightarrow$ GTC
						2. GTC	2. subclinical sz.
12	m	2	Right FLE	Intracerebral bleeding	Yes	1. GTC	1. Complex motor sz. $\rightarrow$ left versive sz. $\rightarrow$ GTC
							2. Subclinical sz.
							3. Dialeptic sz.
13	m	1	Right TLE	Sclerosis of right hippocampus	No	1. Automotor sz.	1. Dialeptic sz. $\rightarrow$ automotor sz. $\rightarrow$ GTC
14	f	1	Left TLE	Sclerosis of left hippocampus	Yes	1. Automotor sz.	1. Vegetative aura $\rightarrow$ automotor sz. $\rightarrow$ aphasic sz. $\rightarrow$ GTC
15	m	5	Right FLE	Unknown	Yes	1. Complex motor sz.	1. Automotor sz./complex motor sz./hyperkinetic sz. $\rightarrow$ GTC
16	f	5	Left TLE	Left temporal Gangliglioma	No	1. Complex motor sz.	1. Epigastric/psychic aura $\rightarrow$ automotor sz. $\rightarrow$ complex motor sz./GTC
17	m	8	Right TLE	Dysplasia DD venous malformation right temporal	Yes	1. Automotor sz.	1. Epigastric aura $\rightarrow$ complex motor sz./automotor sz. $\rightarrow$ clonic sz. of the right face
						2. Automotor sz. $\rightarrow$ complexmotor sz.	
18	m	1	Left TLE	Left temporal dysplasia	No	1. Clonic sz. of the right arm $\rightarrow$ GTC	1. Acoustiv aura $\rightarrow$ automotor sz./right clonic sz./complex motor sz. $\rightarrow$ tonic-clonic sz. of the right face/GTC
19	f	1	Right TLE	Sclerosis of right hippocampus	Yes	1. Automotor sz.	1. Visual aura $\rightarrow$ complex motor sz./automotor sz./aphasic sz./right clonic sz. $\rightarrow$ left versive sz. $\rightarrow$ GTC
							2. Atonic sz.
20	f	9	right TLE	Sclerosis of right hippocampus	No	1. Automotor sz.	1. Epigastric aura/Déjá-vu $\rightarrow$ dialeptic sz./automotor sz. $\rightarrow$ GTC
21	m	5	Left TLE	Left mesial temporal Ganglioglioma	No	1. GTC	1. Unspecific aura $\rightarrow$ dialeptic sz. $\rightarrow$ right versive sz. $\rightarrow$ GTC
22	m	7	Left TLE	Left frontotemporal hemangioma	Yes	1. Bilateral tonic sz.	1. Right somatosensible aura
							2. Right tonic sz.
							3. Clonic sz. of left arm
23	m	22	Right FLE	focal cortical dysplasia right frontal	Yes	1. Complex motor sz.	1. Hyperkinetic sz. $\rightarrow$ GTC
							2. Complex motor sz.
24	m	3	Left TLE	Sclerosis of left hippocampus	Yes	1. Automotor sz.	1. Dialeptic sz. $\rightarrow$ automotor sz. $\rightarrow$ GTC
25	f	2	Left FLE	Unknown	Yes	1. Dialeptic sz. $\rightarrow$ GTC	1. Automotor sz./dialeptic sz. $\rightarrow$ GTC
						2. Automotor sz.	


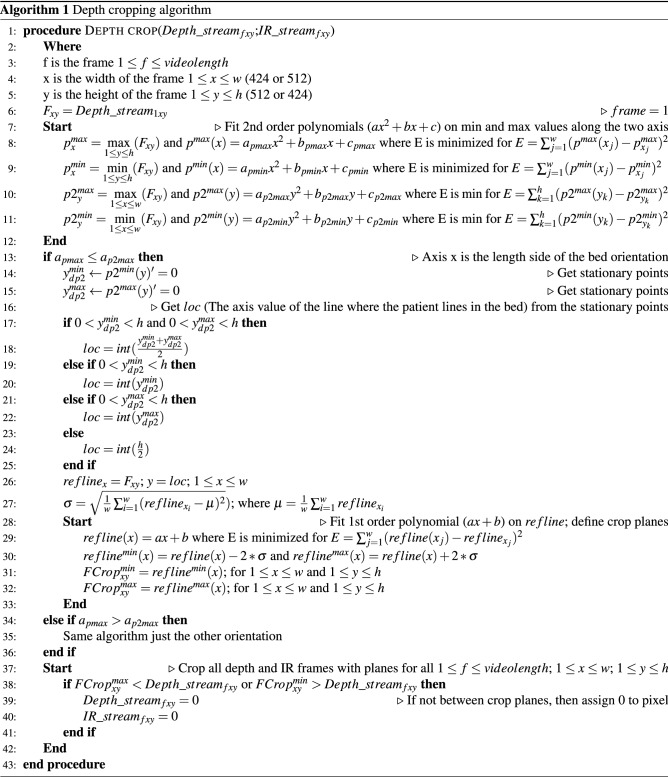
The developed approach is an automatic semi-specialized algorithm, which first identifies the length direction of the bed on the scenery, then determines the distance of interest between 2 planes. Eventually using these two planes it crops the depth and IR videos keeping the pixels only from the required depth volume (Algorithm 1).

The algorithm first fits a 2nd order polynomial on the non-zero minimum and maximum distance values along each axis on the first frame, resulting in four polynomials. From the two polynomial fitted to the non-zero maximum distances from the camera on the x and y axis the one with the smaller 2nd order coefficient defines the length direction of the bed. As an average EMU setting in our dataset tilted approx − 45$^{\circ }$ and mounted on the wall, therefore one axis represents a flat distance as wall-bed with a small fitted curvature, and the other direction represents a floor-bed-floor transition, which produces a larger 2nd order curvature, thus the semi-specialized algorithm. Then the algorithm finds the line where the patient lies in the bed from stationary points of the fitted polynomials. Using this line it defines two parallel planes with an offset from the line. The offset equals to 2 standard deviations of the distance values on the line. Utilizing these planes it crops the depth frames keeping only the volume of interest and then employing the cropped depth frames to mask the IR stream.

It works in most of the EMU settings as most of them has similar arrangement, the camera direction might change, which is handled by the algorithm and then the method locates the person properly and crops the scene.

Video bounding-box cropping In order to further remove unrelated information from the video data, thus maximize classification performance, the seizure videos were automatically cropped as follows. The first frame of each depth cropped IR seizure video was segmented with Mask R-CNN^26^ with a Keras^27^ implementation^28^. The weights of this architecture were pre-trained on MS-COCO dataset, which includes bed and person classes^29^. This automated segmentation provides the bounding boxes of all persons and beds detected on the first frame. Then the bed and person bounding box with the highest confidence is selected and a merged bounding box is created. This resulting bounding box was expanded with +30% in all direction to account for violent movements through the seizure, which was then used to crop automatically the whole video sequence (dataset B, B0.5).

With the aim of evaluating the influence of the depth cropping algorithm on the classification performance this detected bounding box was utilized also to crop the raw IR footage as well for dataset A. This method ensured the only difference between dataset A and B to be the utilization of the depth cropping algorithm, as the bounding boxes were the same.

A visual quality check of 5 frames from each video distributed evenly through the length of of the sequence including the first and the last frames of each 115 videos was performed (see results).

#### Pre-processing for feature extraction

To comply with the I3D input requirements^24^ the cropped videos were converted from one channel gray (uint8) to RGB (uint8) representation. These were then resized with preserving the aspect ratio of the videos to have the largest dimension of the frames as 224 pixels with bilinear interpolation and then padded to 224 $\times$ 224 pixels. The pixel values were rescaled between − 1.0 and 1.0 as in Eq. (1).

1 $$\begin{aligned} frames_{rescaled}=\frac{2*frames}{255.0}-1 \end{aligned}$$

#### Temporal slicing and creating samples

The temporal slicing strategy to create samples considers both clinical and technical aspects.

From the clinical point of view MOIs are significantly shorter than the seizure length, which provide clinical diagnostic value already with shorter sequences. These movements are a complex combination of posing, speed, frequency and path of the movements, which can be repetitive actions, such as automatisms. Thus, part of a seizure or MOI may already be as definitive as the whole seizure or MOI would be. From the clinical experience of our research group we know that these MOIs have in case of TLE seizures typically frequencies within 0,4 [Hz] to 1,8 [Hz], and in case of FLE seizures these movements are slightly faster^30^. Therefore shorter sections of the seizure, in our case 60 frames, may already be used to differentiate between our described 3 classes. Additionally, several MOIs are present during seizures in different sequences, therefore this method helps to avoid the overfit for the sequence of MOIs, rather enforces the algorithm to recognize clinically relevant movements from shorter sequences, which differentiate seizures.

From the technical aspect, due to the class imbalance and the limitation of the available data the seizure videos were temporally sliced to 60 frame samples. The leftover frames at the end of the seizures, which were less than 60 frames, but more than 9 were also included as samples. The dataset is highly imbalanced between the number of FLE and TLE seizures, however number of frames representing the classes are more balanced, as there is almost half of TLE seizures, than FLE but with more than the double of average length. Utilizing this temporal slicing technique the number of samples per seizure class were almost balanced out. During training the GPU RAM is a limiting factor, which has to accommodate a considerable size of batch from the video samples to learn to classify relevant features, as these MOIs are not necessarily included in all of the seizures. Thus, to have a good convergence of training on these diverse features, this method assists in achieving ideal data distribution for training, both inside the batches and between classes. Moreover this strategy is advantageous as the feature extraction I3D architecture^24^ was optimized for a similar temporal receptive field, exactly 64 frame samples from the 10 s action videos.

This temporal slicing strategy resulted in 1252 FLE, 1372 TLE and 8852 Prepost samples (Table 3a). With aim of evaluating temporal augmentation a sub-dataset was extracted with 50% overlap of the snippets (Table 3a). An overview of the three type of preprocessing for the sub-datasets is presented in Table 3b.

### Feature extraction

Features were extracted with a Keras implementation^31^ of Inflated 3D Convnet (I3D)^24^. I3D is designed for human action recognition, therefore it is especially suitable for spatio-temporal feature extraction of human movements. The architecture is based on Inception-V1^32^, with pre-training on ImageNet^33^. Then the I3D architecture was trained on the Kinetics-400 dataset^34^, which consists of 400 human action classes and over 400 clips per class^24^.

For feature extraction the last classification layers were removed (Fig. 3) and the 9–60 frame length samples were evaluated with the network. This resulted in a 7 timestep feature vector with 1024 features per timestep. Samples with less than 60 frames, however yielded less than 7 timesteps, thus these were pre-padded with dummy feature vectors to match the dimensions (7 $\times$ 1024), with a values of − 1 for future masking.

### Implemented classifier architectures

#### LSTM classifier architecture

Classification was carried out with a long short-term memory (LSTM) based classifier, to further exploit temporal features (Fig. 3). Preceding the first LSTM layer a masking layer hid the dummy − 1 value inputs, excluding them from the optimization process. Afterwards the classifier composed of two LSTM, two FC layers and a prediction layer (Fig. 3).

Employing Talos^35^, random hyperparameter optimization was used to determine the number of units in the LSTM [32, 256] and fully connected (FC) [16, 128] layers, the loss function [mean squared error, binary cross-entropy], the dropout (DO) rate [0, 0.5], the recurrent DO rate [0, 0.5], the number of epochs [50, 2000], and the batch size [50, 2000].

The hyperparameter search pointed to the utilization of 128 and 64 units for the LSTM layers and two fully connected (FC) layers, with 64 and 32 units. Except for the temporally augmented 3 class case (B0.5) were it lead to 128 and 128 units for the LSTM and 128 and 64 units for the FC layers (extended LSTM architecture). Batch normalization (BN) and dropout (DO) were used as regularization layers , with a dropout rate of 0.5. Dropout was applied after batch normalization to prevent variance shift when transferring the model from training to test state^36^. Recurrent dropout was applied with a dropout rate of 0.3. L2 regularization was applied on the kernels of the dense layers and LSTM layers. Furthermore, the LSTM layers recurrent regularization was also L2. Moreover, activity regularizer were applied to the LSTM layers and bias regularization to the FC layers. ReLU activation was used on the dense layers. The kernels of the FC layers were initialized with He uniform initializer^37^. Weighted mean squared error was used for the 2 class and categorical cross-entropy for the 3 class case as loss function. It was optimized with Adam optimizer, using previously described hyperparameters^38^ (learning rate of 0.001, $\beta _1=0.9$ and $\beta _2=0.999$)^38^. The last classification layer was one unit with sigmoid activation for the 2 class case and three units with a softmax activation for the 3 class. The full proposed system is illustrated on Fig. 3.

#### I3D classifier

In order to provide a baseline of the developed approach the original I3D classifier layers^24^ were also implemented and retrained for our dataset (Fig. 3). Weighted categorical cross-entropy was used as loss function and the architecture was trained with SGD optimizer with 0.9 momentum, as in the original I3D paper^24^.

#### Training methods

The architectures were trained for 2000 epochs with a batch size of 500 and 1000 samples for the 2 class and 3 class case respectively. Early stopping was used, to ensure generalization, thus the architecture with the highest f1 validation score was used.

All pre-processing, computation and training were carried out with a PC, equipped with 2 NVIDIA GeForce GTX 1080 Ti (2*11GB) video cards in SLI configuration, an i7-6700K CPU and 64 GB RAM.

### Cross-validation

To ensure the training of a generalized classifier, seizures were grouped by patients. It was essential to prevent data-leakage, such as non-epileptic related subject specific features in training and validation set at the same time. Moreover, it is advantageous to avoid overfitting the classifier even to epilepsy related but subject specific features as well, thus improving generalization of the classifier.

To confirm the performance of the classifiers a 5-fold cross-validation was performed. In each fold 12 FLE and 8 TLE subjects were in the training set and 3 FLE and 3 TLE subjects in the validation set. This sorting of the seizures however increased class imbalance, as the available number of seizures per patient varies a lot in the dataset. Thus, the temporal slicing of seizures (“Data pre-processing”) and weighted class handling both in training (“Implemented classifier architectures”) and evaluation phase (“Classification of I3D features”) were used to address this issue.

## Supplementary Information

Supplementary Information.

## Data Availability

The data that support the findings of this study are available on request from the corresponding author, J.P.S.C. The data are not publicly available due to containing information that could compromise the privacy of research participants.
