# Supplementary material for: Novel 3D video action recognition deep learning approach for near real time epileptic seizure classification
Source: Sci Rep. 2022 Nov 15;12:19571. doi: 10.1038/s41598-022-23133-9 (PMC9666544; doi:10.1038/s41598-022-23133-9)
Supplement: Supplementary file 1 — Supplementary Information. [file 41598_2022_23133_MOESM1_ESM.pdf]

1 Additional information

1.1 Confusion matrices sample normalized

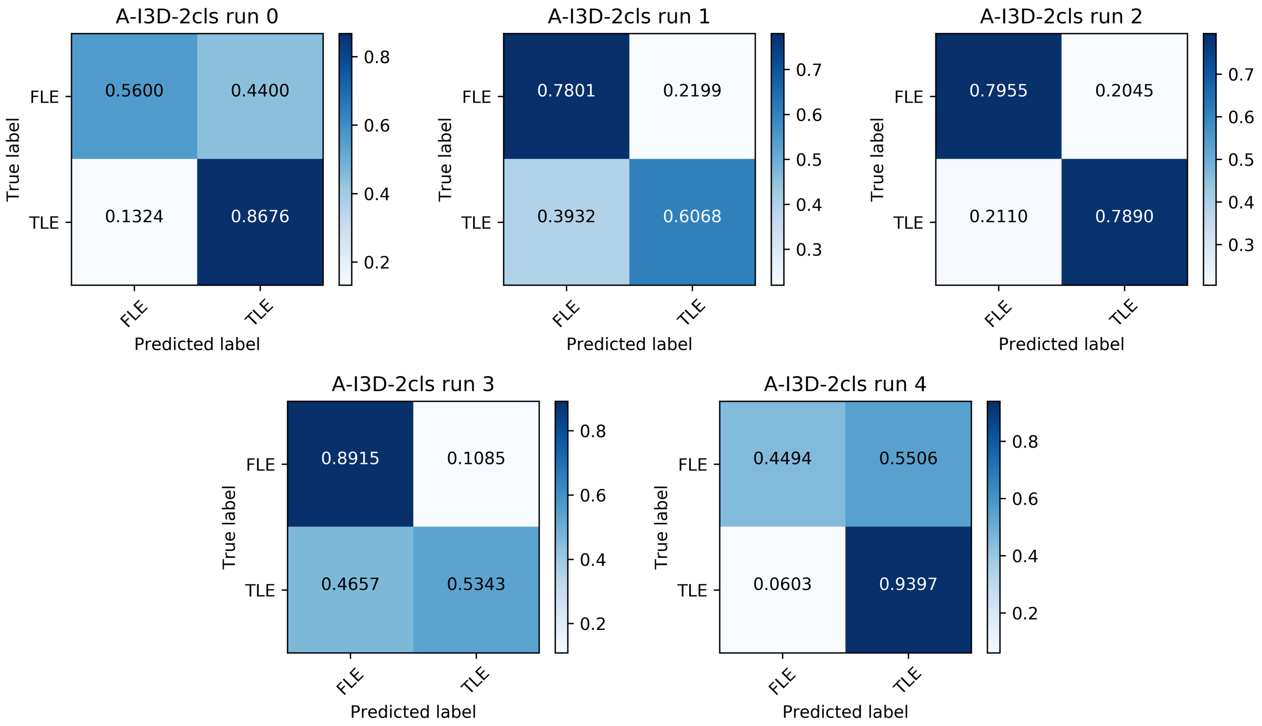

(a) A-I3D-2cls normalized

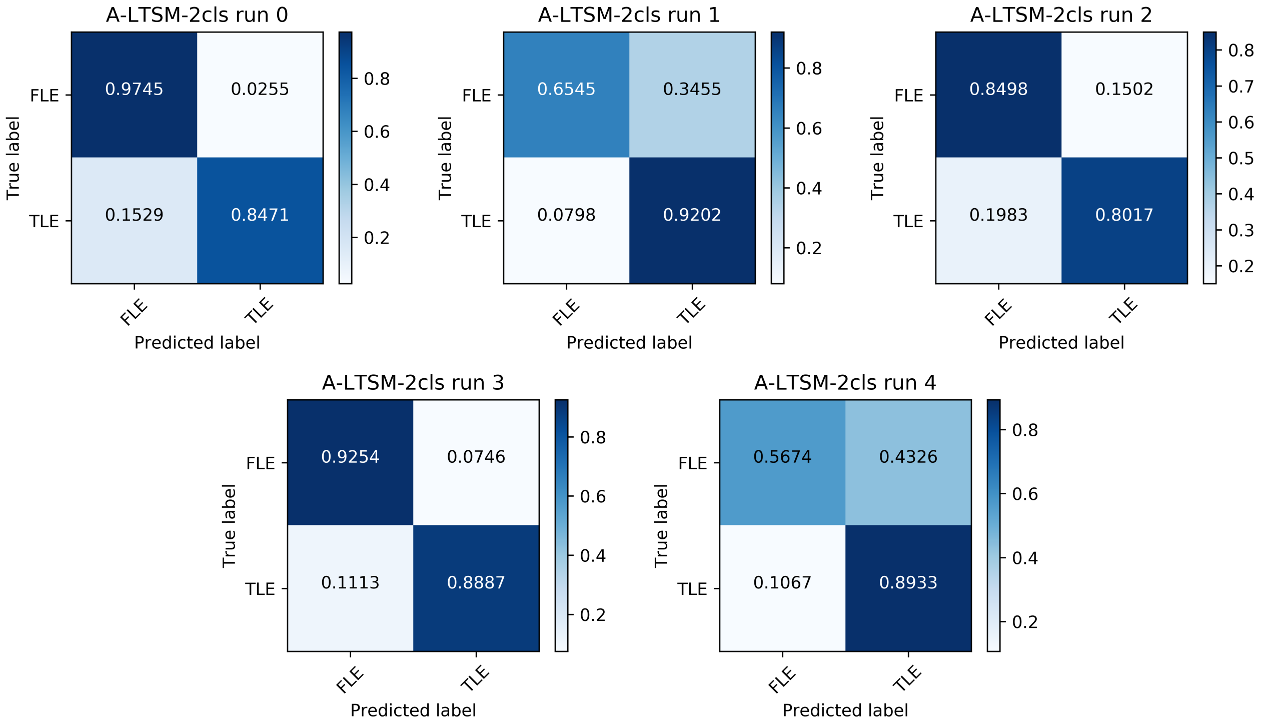

(b) A-LSTM-2cls normalized

Figure A.1. Dataset A, 2 classes, normalized

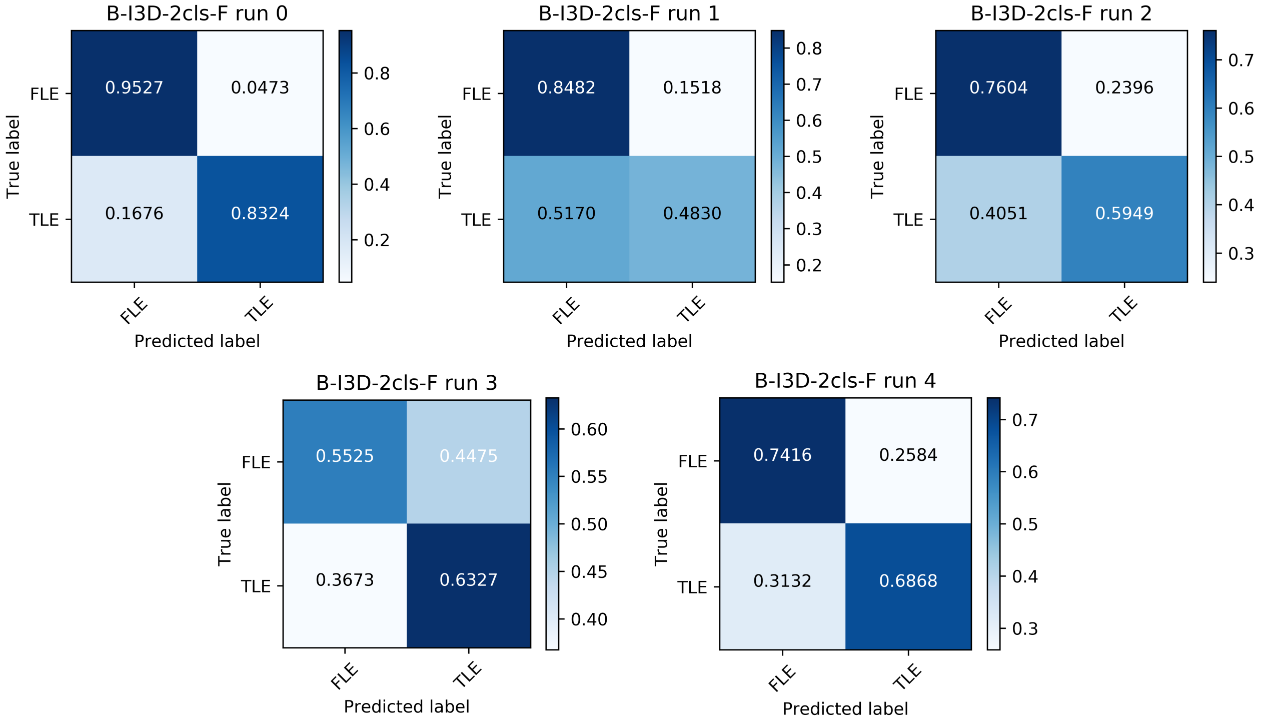

(a) B-I3D-2cls normalized

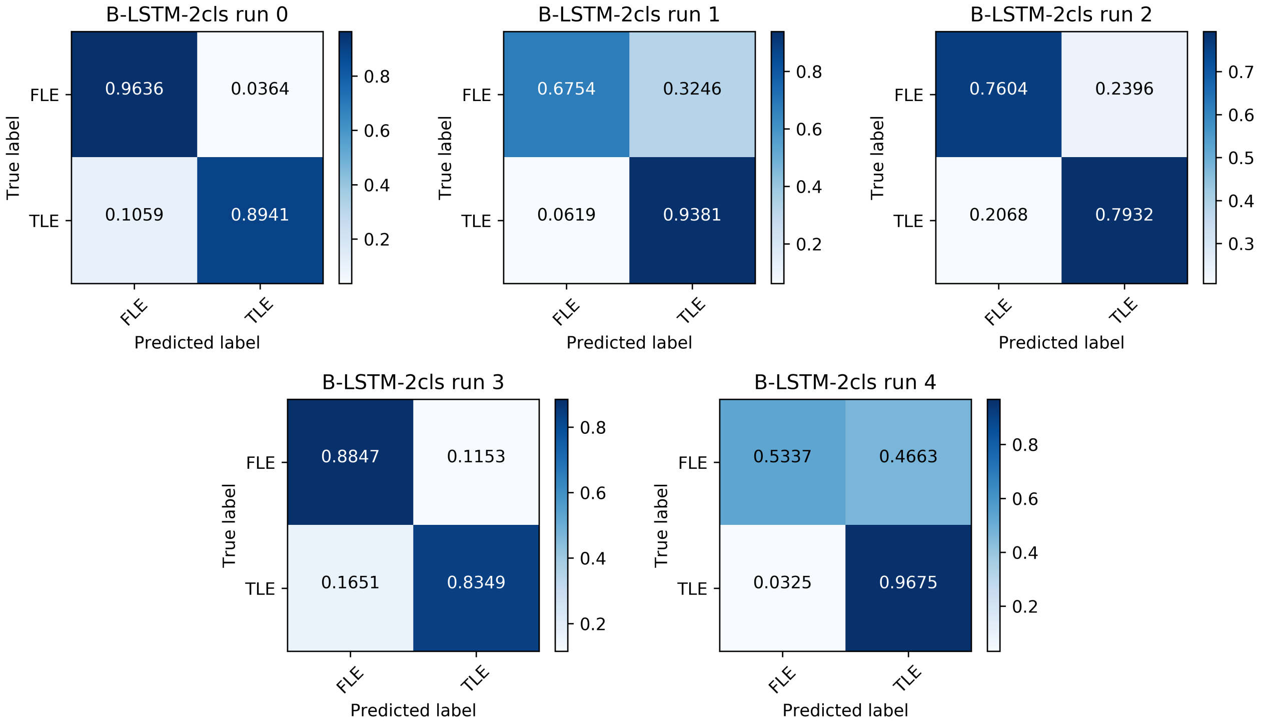

(b) B-LSTM-2cls normalized

Figure A.2. Dataset B, 2 classes, normalized

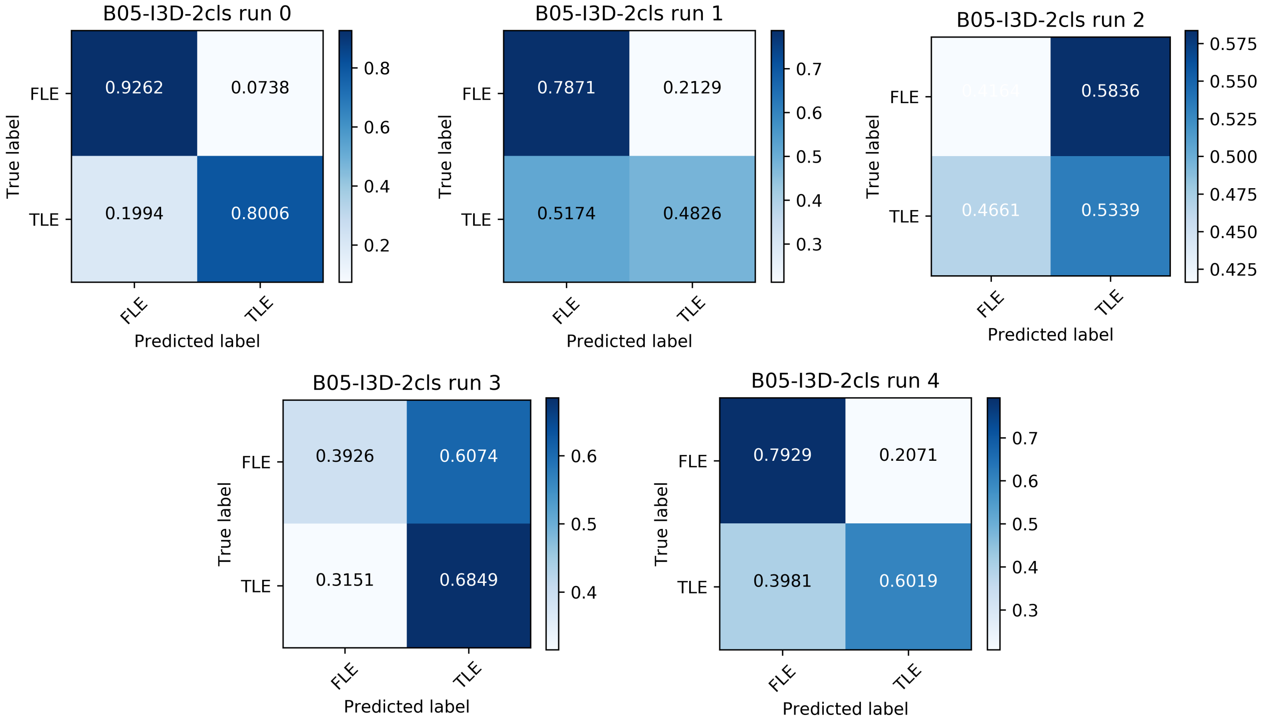

(a) B05-I3D-2cls normalized

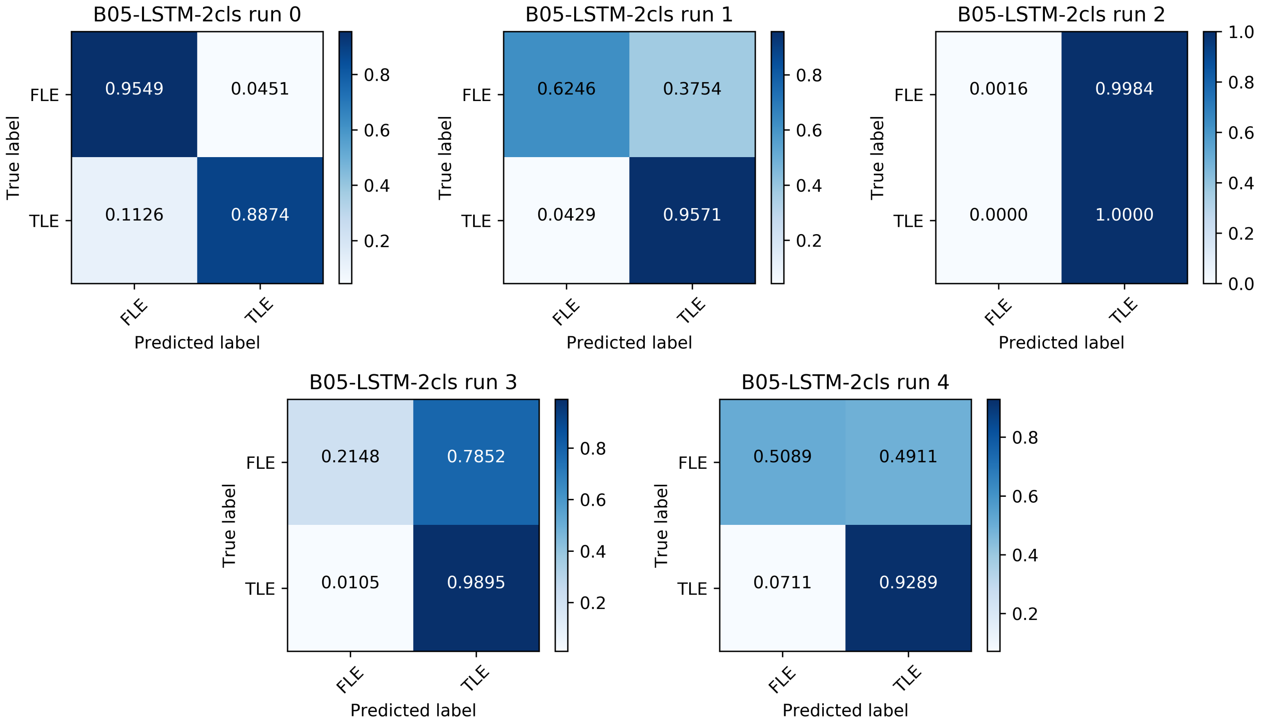

(b) B05-LSTM-2cls normalized

Figure A.3. Dataset B05, 2 classes, normalized

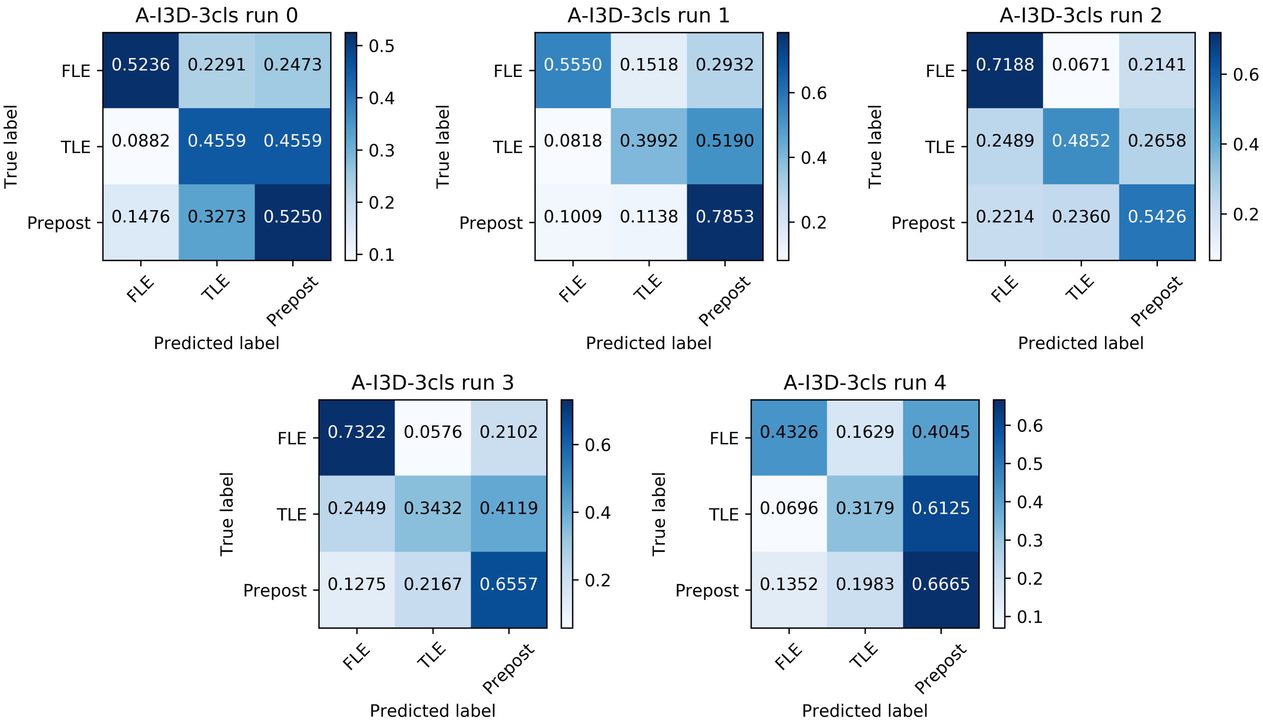

(a) A-I3D-3cls normalized

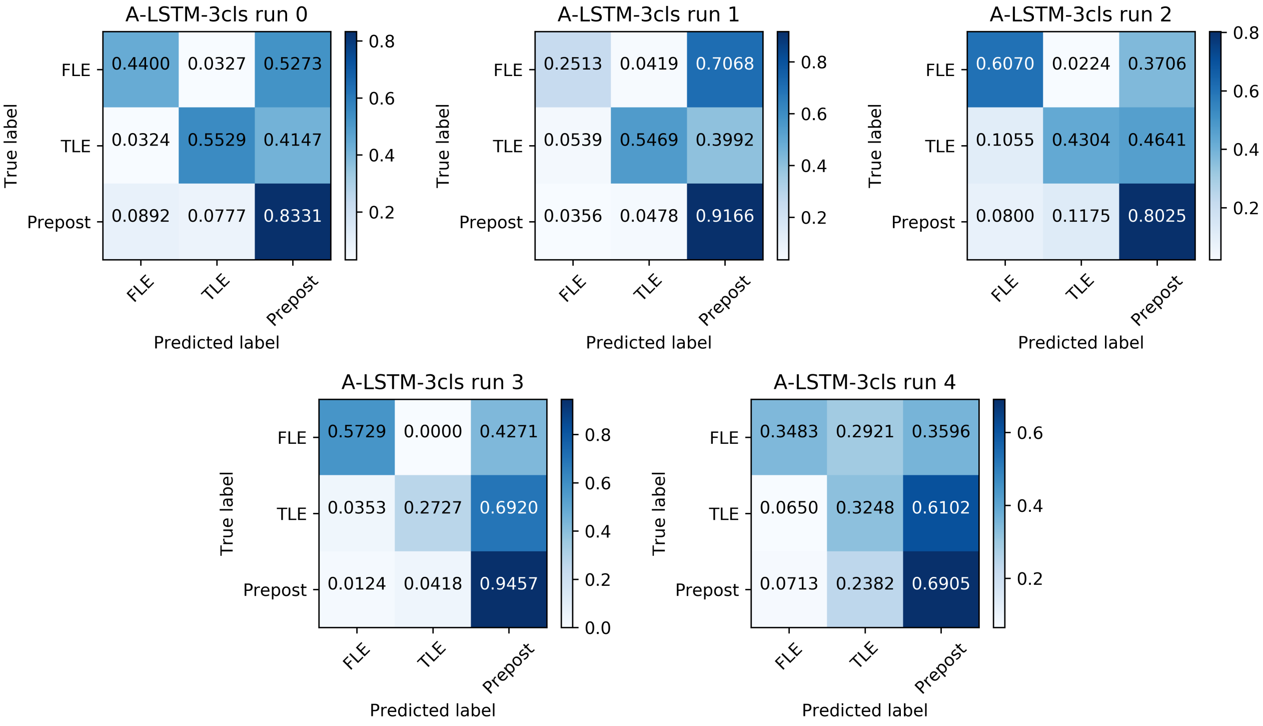

(b) A-LSTM-3cls normalized

Figure A.4. Dataset A, 3 classes, normalized

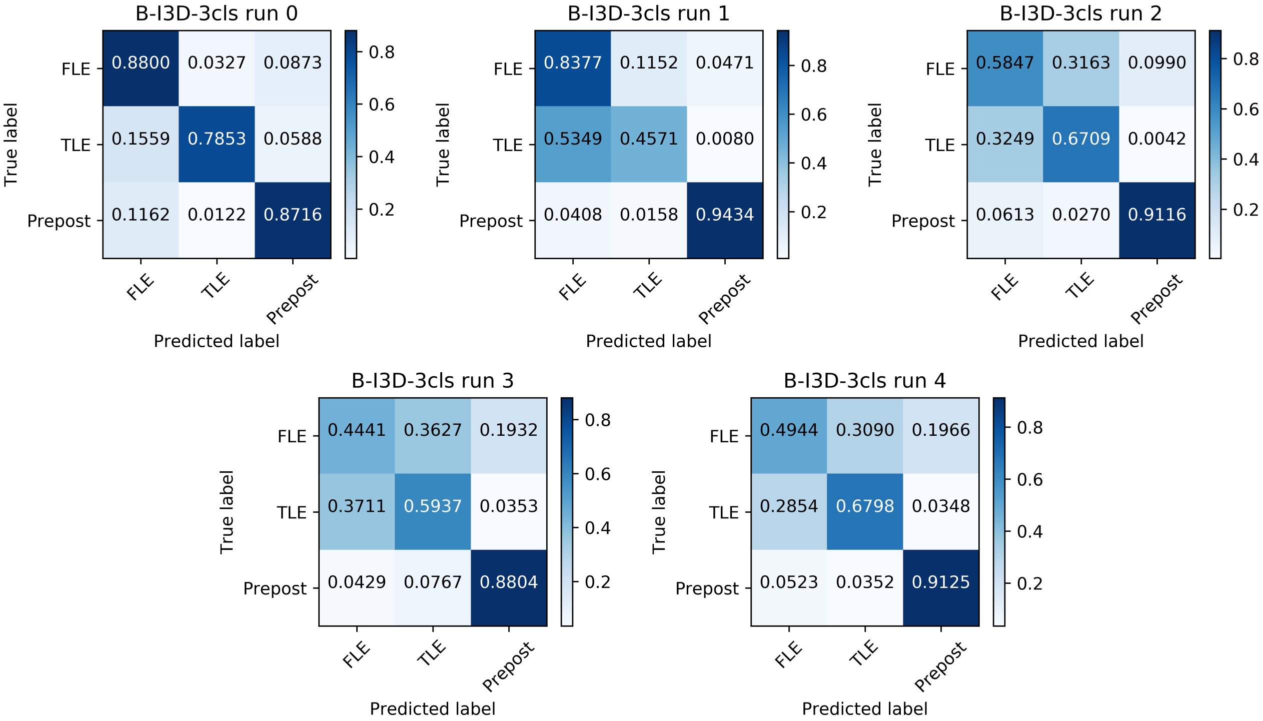

(a) B-I3D-3cls normalized

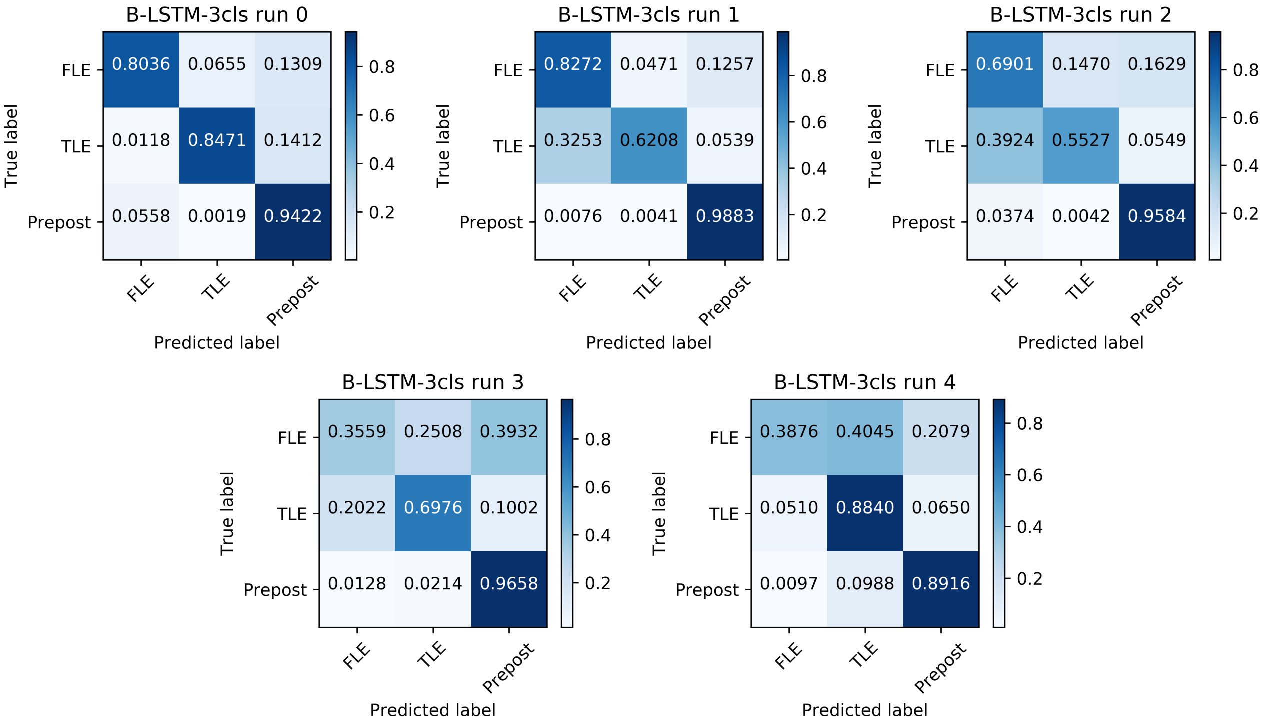

(b) B-LSTM-3cls normalized

Figure A.5. Dataset B, 3 classes, normalized

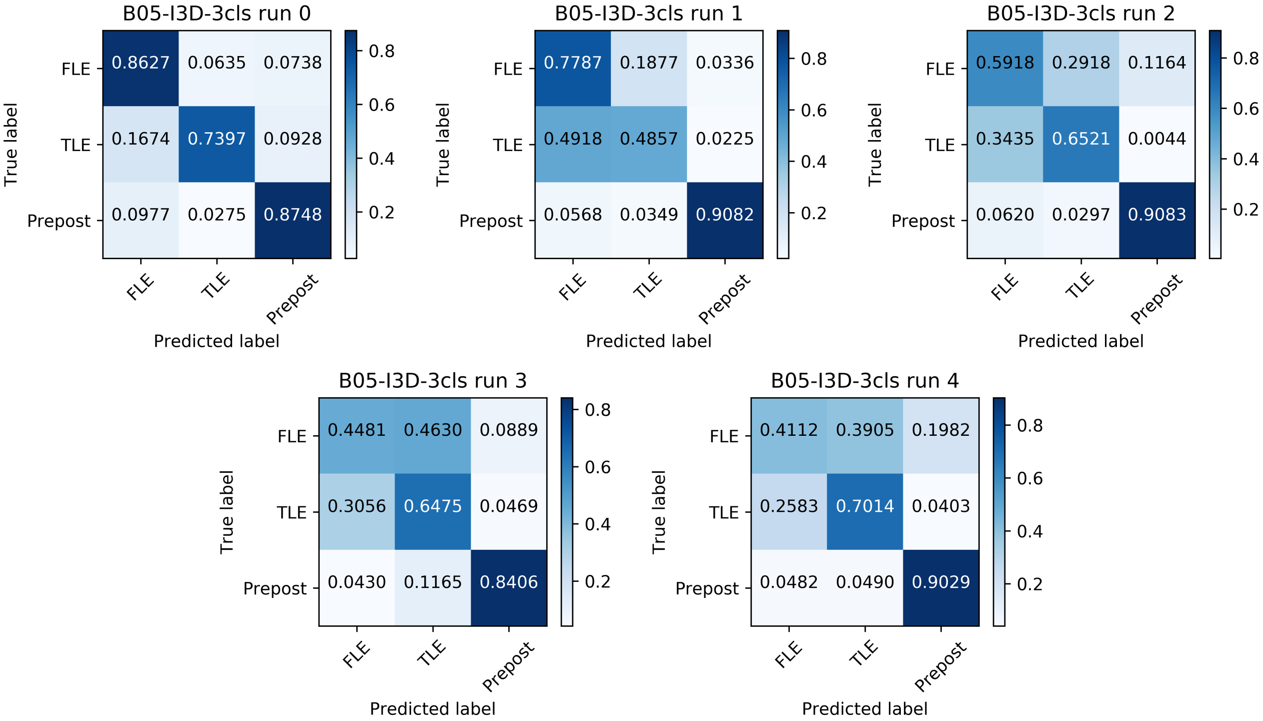

(a) B05-I3D-3cls normalized

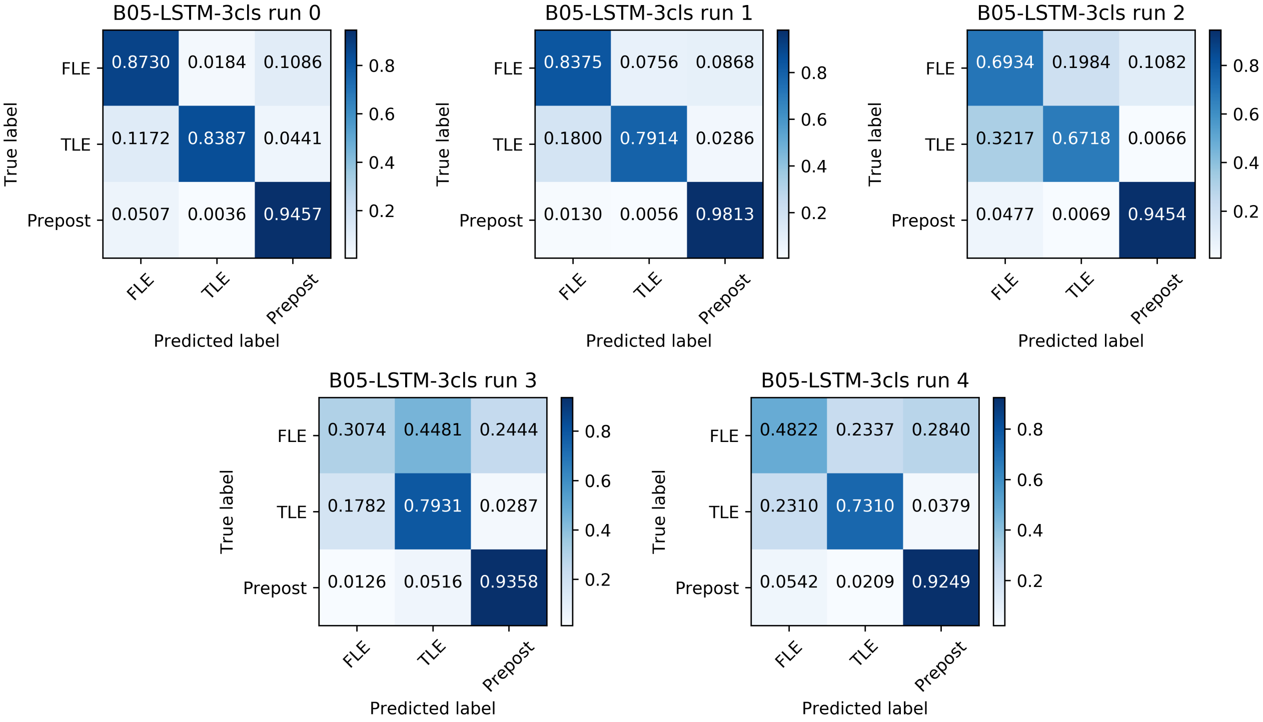

(b) B05-LSTM-3cls normalized

Figure A.6. Dataset B05, 3 classes, normalized

1.2 Confusion matrices, number of samples

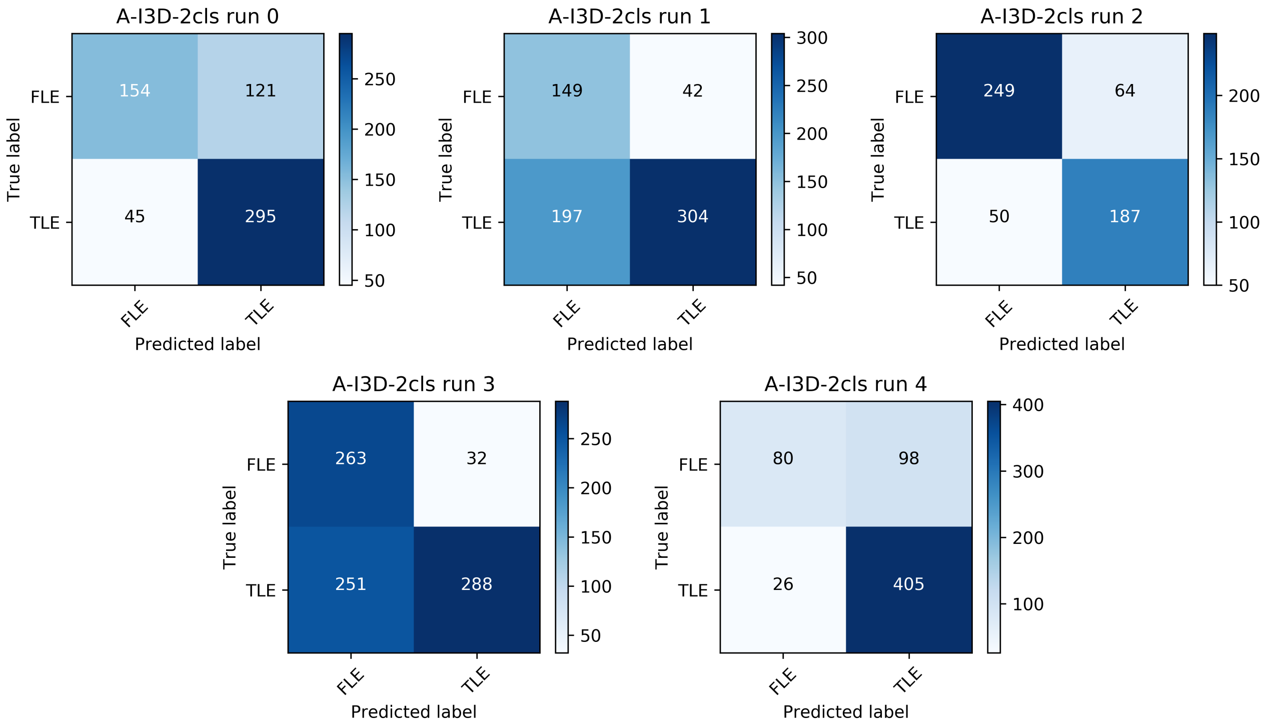

(a) A-I3D-2cls

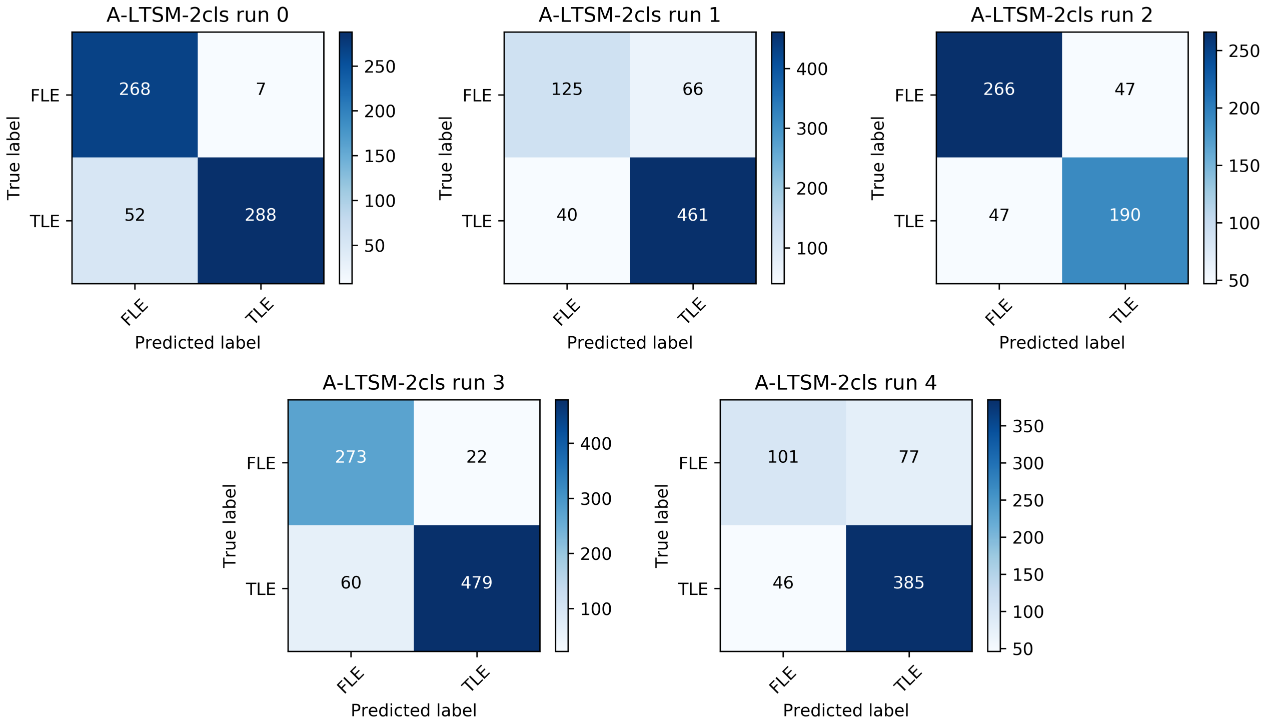

(b) A-LSTM-2cls

Figure A.7. Dataset A, 2 classes

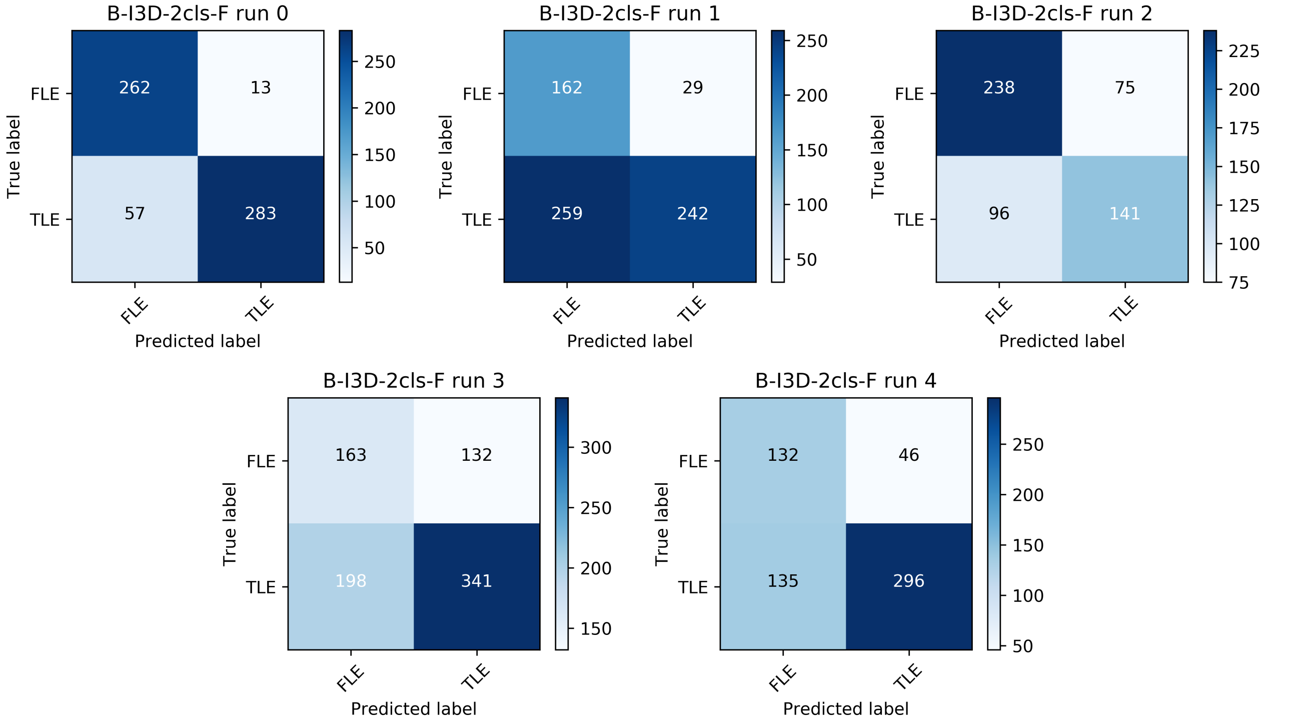

(a) B-I3D-2cls

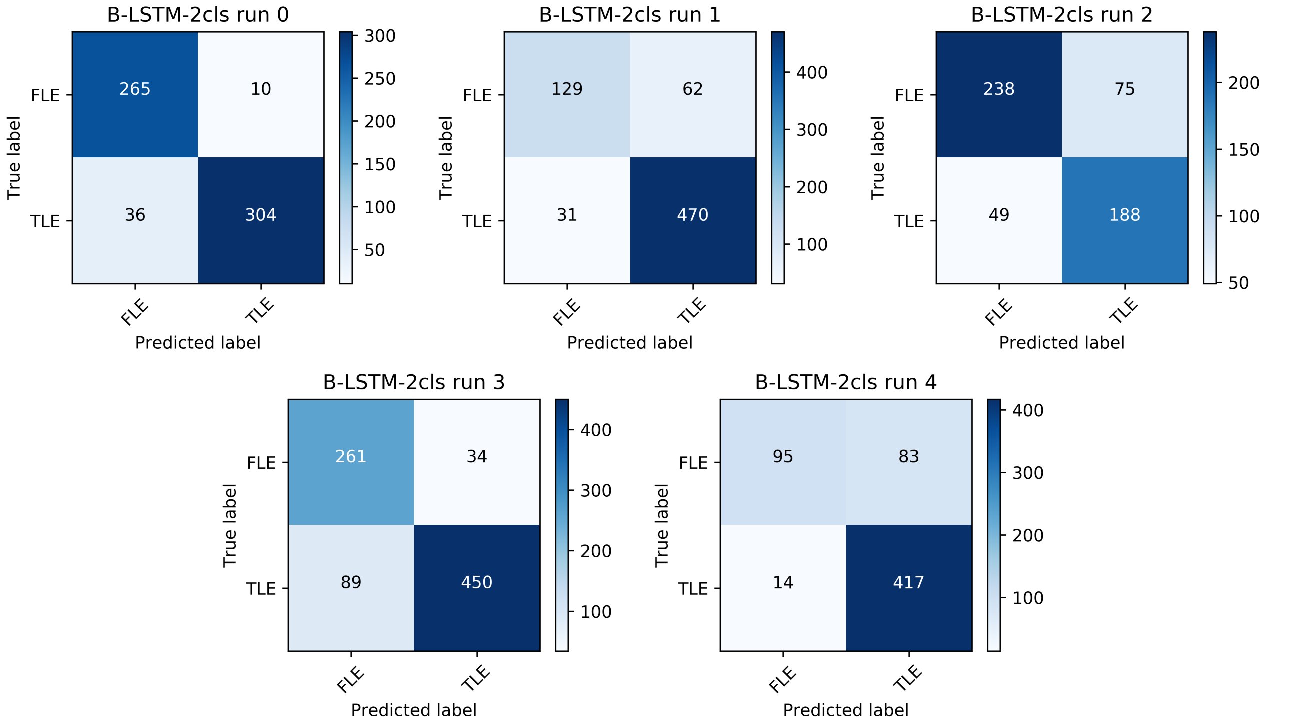

(b) B-LSTM-2cls

Figure A.8. Dataset B, 2 classes

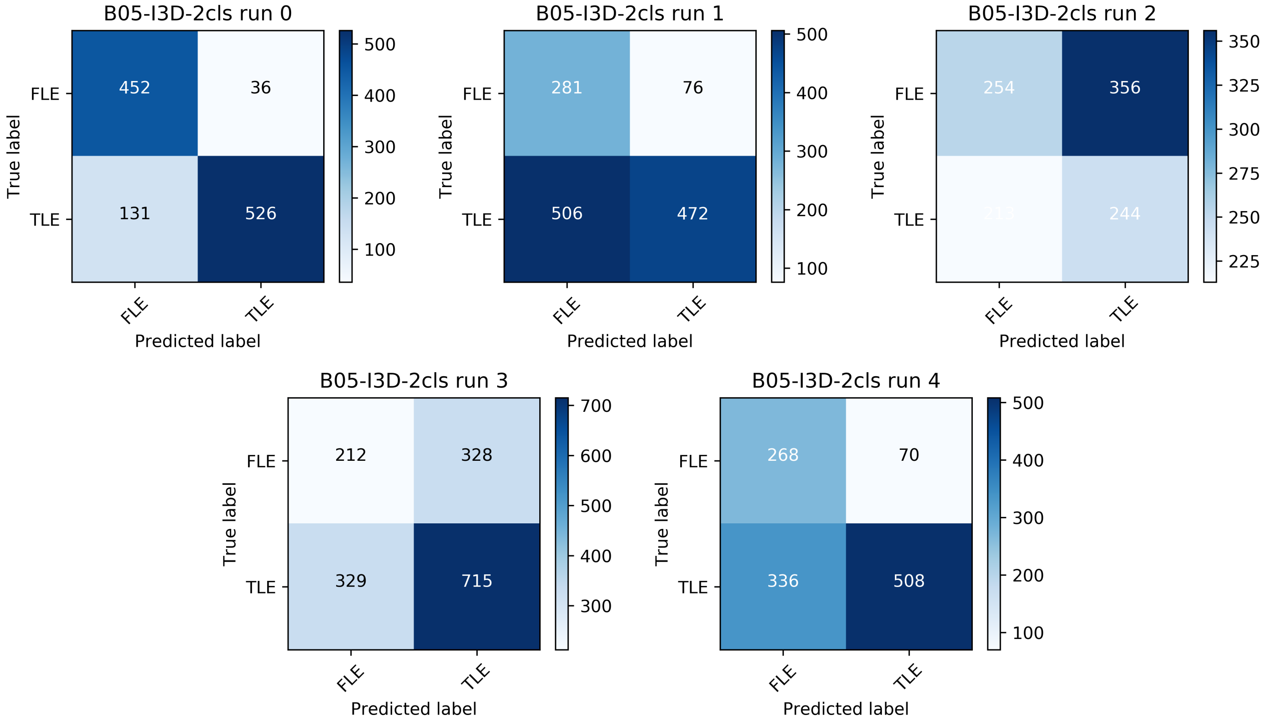

(a) B05-I3D-2cls

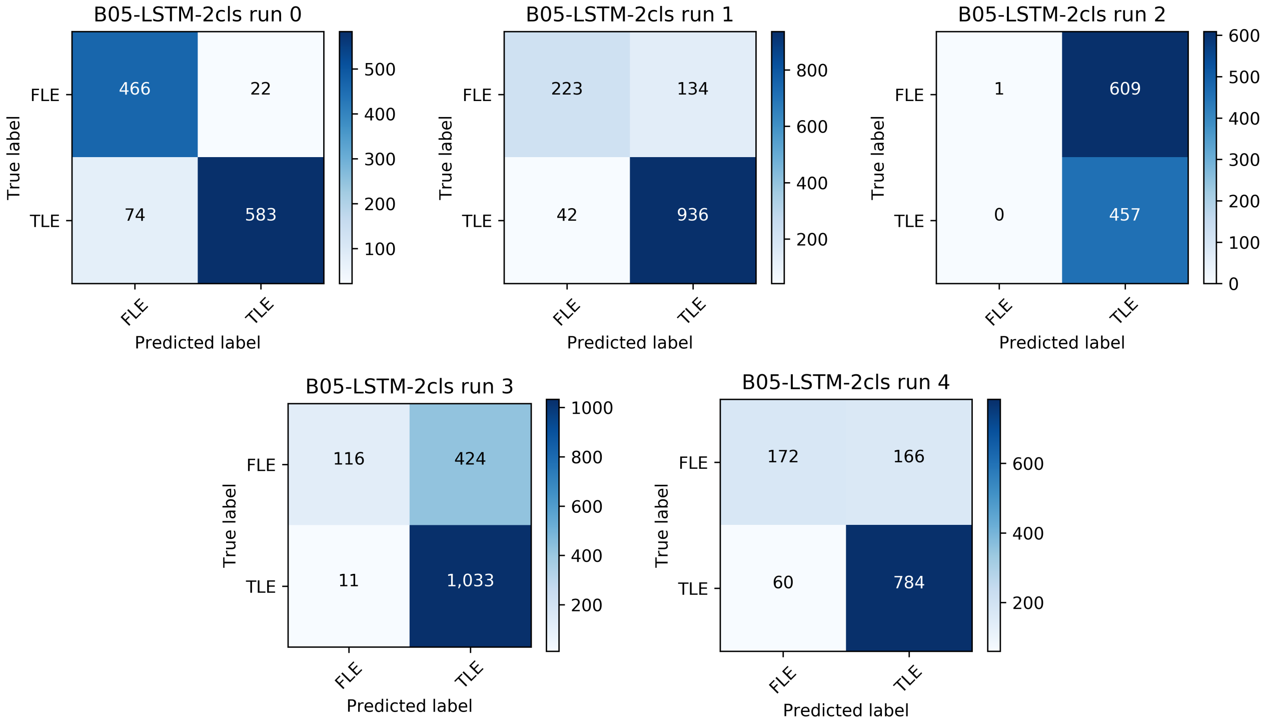

(b) B05-LSTM-2cls

Figure A.9. Dataset B05, 2 classes

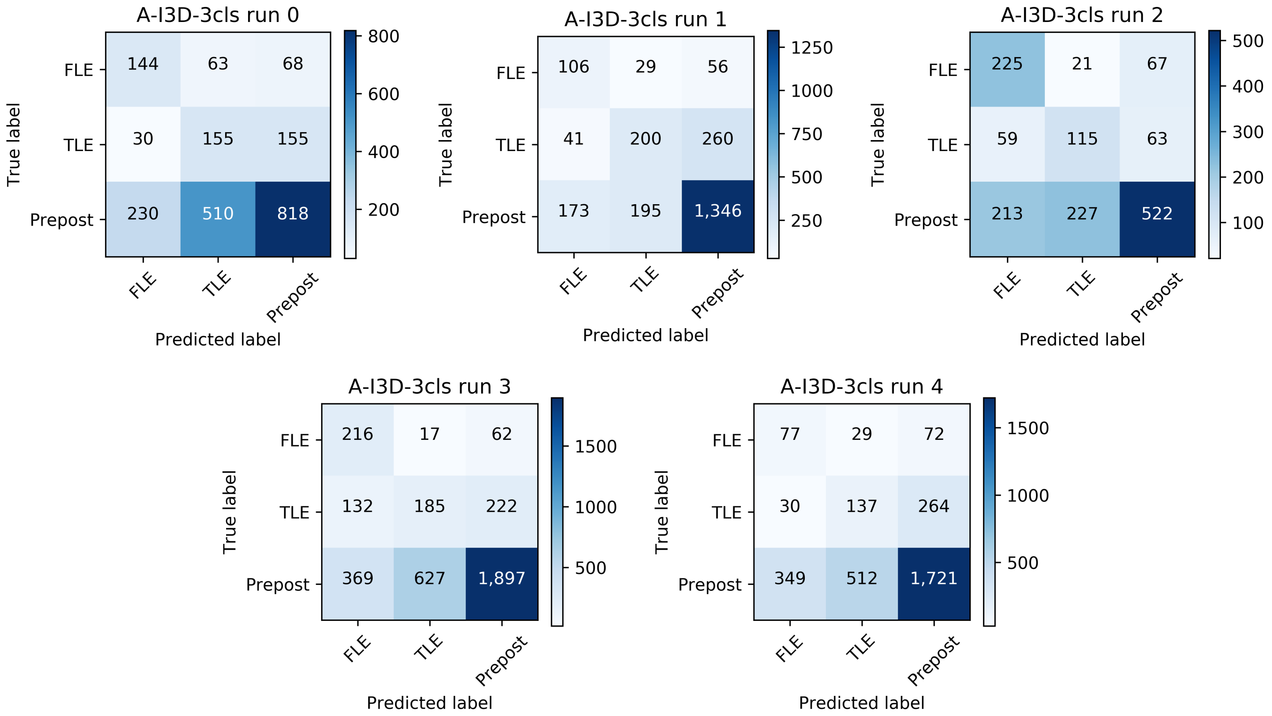

(a) A-I3D-3cls

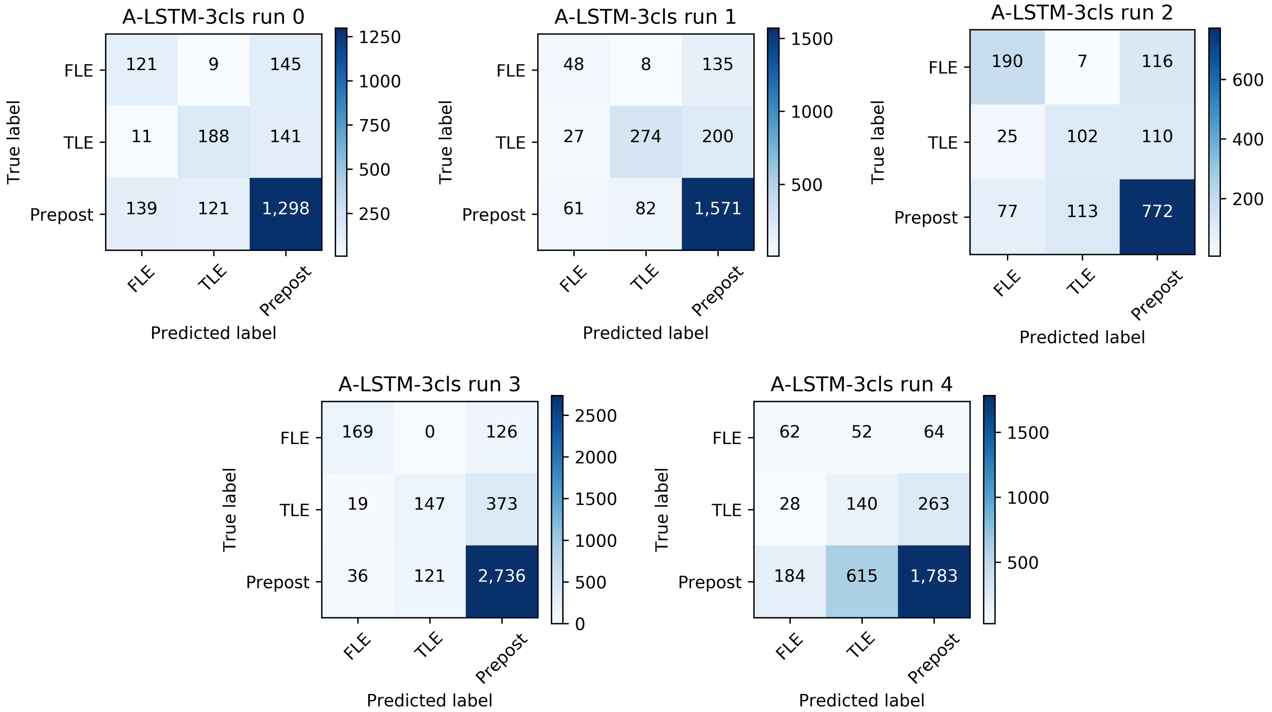

(b) A-LSTM-3cls

Figure A.10. Dataset A, 3 classes

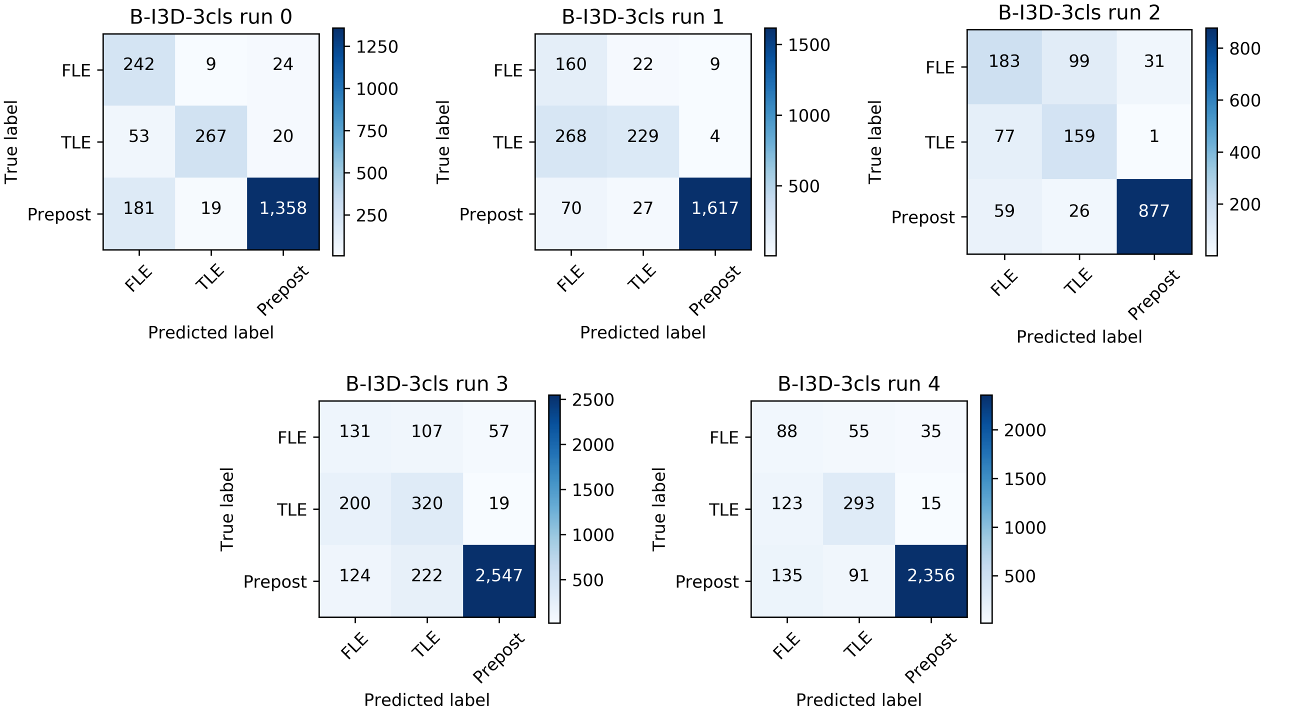

(a) B-I3D-3cls

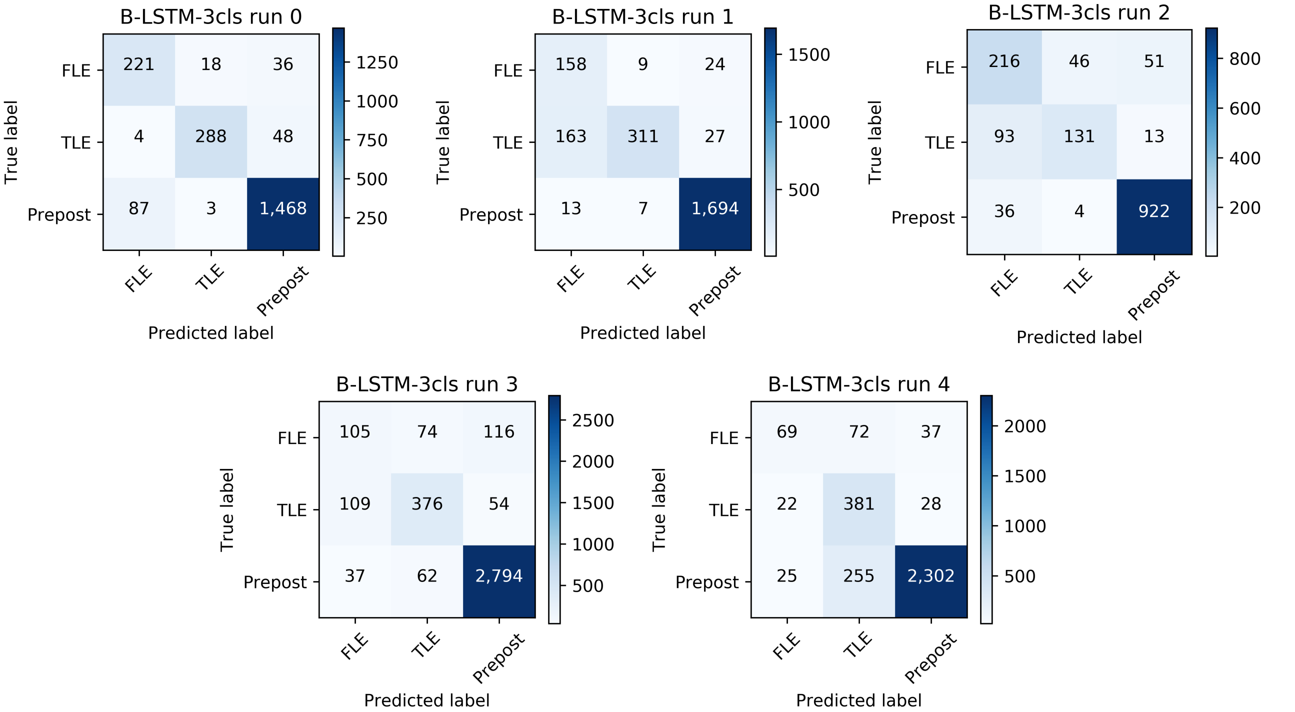

(b) B-LSTM-3cls

Figure A.11. Dataset B, 3 classes

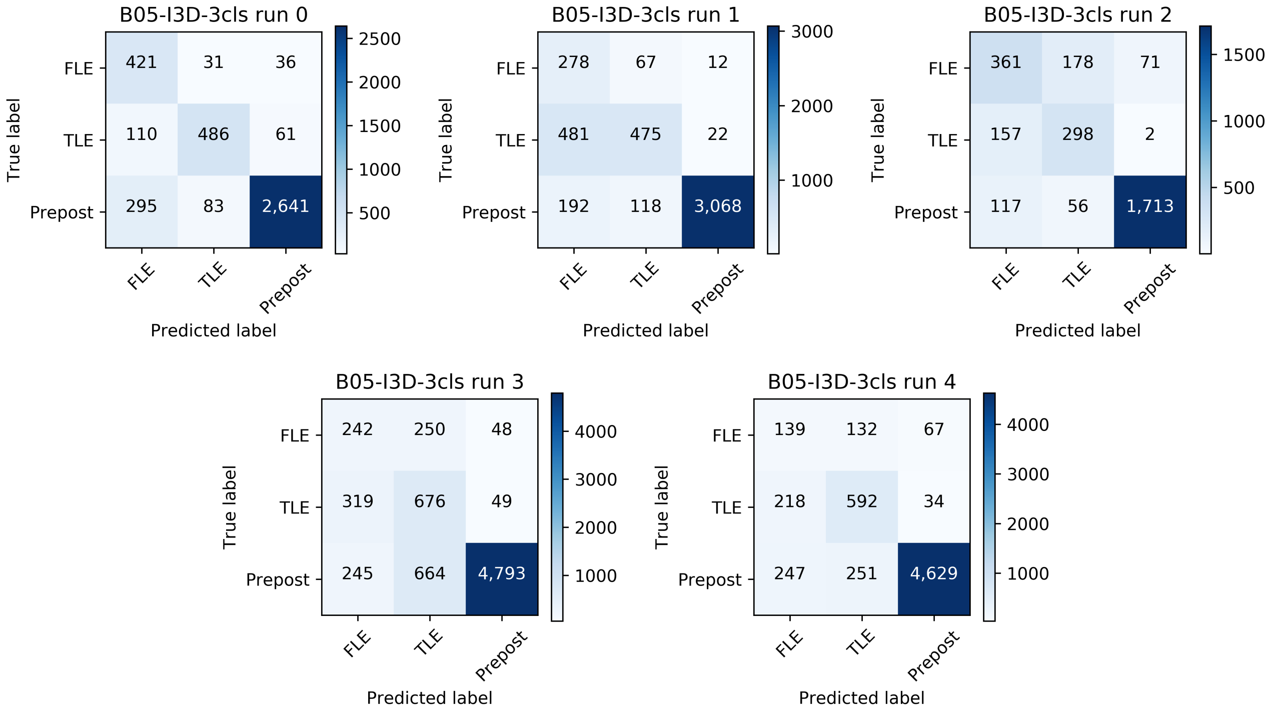

(a) B05-I3D-3cls

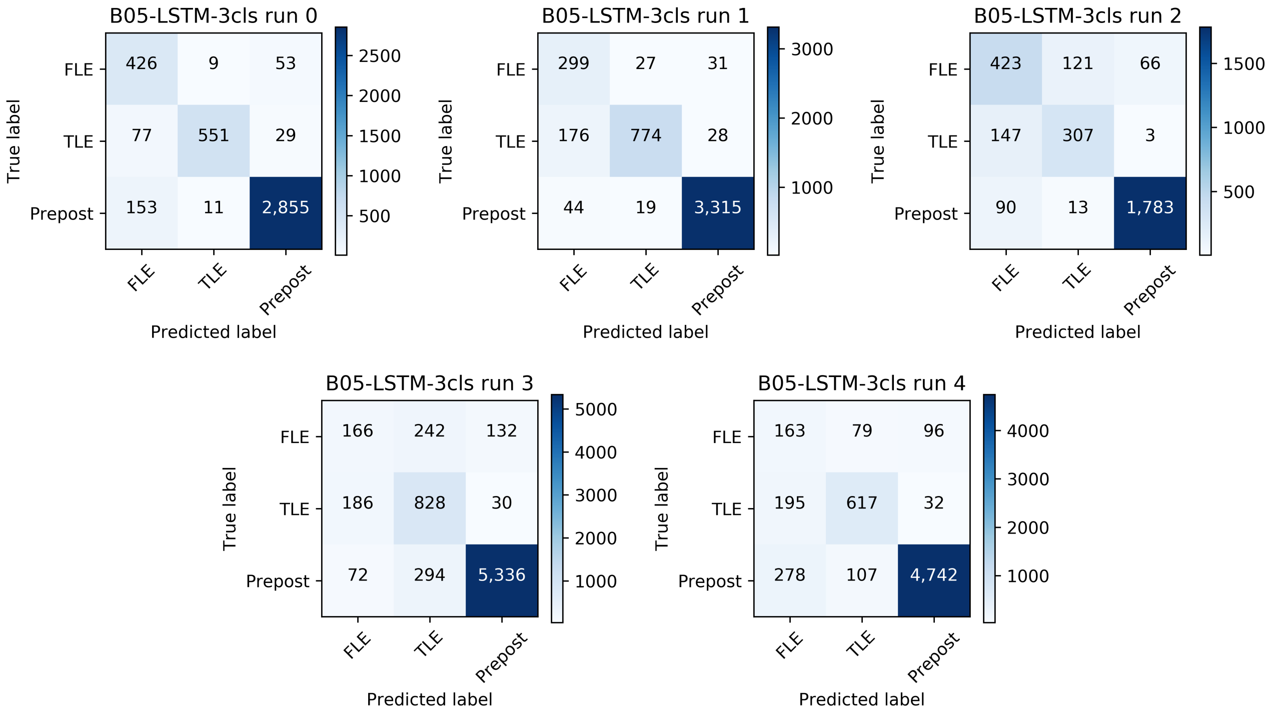

(b) B05-LSTM-3cls

Figure A.12. Dataset B05, 3 classes
